# The E2 ubiquitin‐conjugating enzymes OsUBC11 and OsUBC12 promote rice seed germination by ubiquitinating and destabilizing OsABI3 in the ABA signaling pathway

**DOI:** 10.1111/tpj.71096

**Published:** 2026-09-10

**Authors:** Tian‐Ci Xie, Tao Qing, Chuang Yang, Jia‐Mu Wang, Hai‐Ping Lu, Jian‐Xiang Liu

**Affiliations:** ^1^ State Key Laboratory of Plant Environmental Resilience, College of Life Sciences Zhejiang University Hangzhou 310027 China

**Keywords:** rice (*Oryza sativa*), ubiquitin‐conjugating enzyme, OsUBC11/12, ABA signaling, OsABI3, seed germination, salt stress

## Abstract

The ubiquitin‐conjugating enzymes (E2) play critical roles in plant stress responses and development, but their functions in ABA‐mediated seed germination in rice remain largely unknown. We identified two homologous E2 enzymes, OsUBC11 and OsUBC12, which are highly expressed in rice seeds and induced by multiple abiotic stresses including salt, osmotic stress, and ABA treatment. We demonstrated that OsUBC11 and OsUBC12 positively regulate rice seed germination, especially under NaCl and ABA treatment, as the double mutants exhibited delayed germination and hypersensitivity to ABA and salt, while overexpression lines showed accelerated germination and reduced sensitivity. OsUBC11 and OsUBC12 negatively regulate the expression of ABA‐responsive genes, including *OsABI3*, *OsABI5*, *OsRAB21*, and *OsLEA3*. Protein interaction assays demonstrated that OsUBC11/12 directly interact with OsABI3, a key transcription factor in ABA signaling. OsUBC11/12 possess ubiquitin‐conjugating activity and promote the ubiquitination and subsequent degradation of OsABI3, thereby reducing OsABI3 protein stability. Together, our findings establish that OsUBC11 and OsUBC12 positively regulate seed germination under salt stress by ubiquitinating and destabilizing OsABI3, thus attenuating ABA signaling.

## Significance Statement

This work reveals that two rice ubiquitin‐conjugating enzymes, OsUBC11 and OsUBC12, promote seed germination under salt stress by promoting degradation of the ABA signaling factor OsABI3. The findings uncover a crucial mechanism for attenuating ABA responses, offering potential targets for improving crop germination under stressful conditions.

## INTRODUCTION

Throughout their growth and development, plants are continuously threatened by various biotic and abiotic stresses. Adverse environmental factors such as low temperature, high temperature, salinity, and drought restrict plant growth and even endanger plant survival (Zhu, 2016). Among these, soil salinization is one of the most common abiotic stresses affecting crop growth, development, and yield. Human activities and climate change have exacerbated land salinization, leading to reduced agricultural productivity, which sharply conflicts with the increasing global demand for food (Ismail & Horie, 2017). A recent study showed that irrigation with saline water reduces average crop yield by 21.99% compared to freshwater irrigation, resulting in substantial economic losses (Qiao et al., 2026). Rice is one of the most important staple crops and is among the most sensitive major cereals to salt stress. Field experiments under saline conditions have shown that salt stress reduces rice yield by as much as 35–50% (Wei et al., 2024). Therefore, elucidating the molecular mechanisms underlying responses to salt stress in plants such as rice is of great theoretical and practical significance.

In the complex regulatory network of plant responses to salt stress, phytohormones play a central role. Among them, abscisic acid (ABA) is recognized as the most important stress hormone, playing a critical role particularly in salt stress responses (Chen et al., 2024). ABA acts as a central integrator that reprograms plant developmental processes and links them to adaptive signaling cascades under salt stress, especially osmotic stress (Golldack et al., 2014). Studies have shown that the OsGAPC1‐OsSGL module negatively regulates rice salt tolerance by mediating ABA biosynthesis (Jiang et al., 2024). Under salt stress, endogenous ABA levels rapidly increase, enhancing ABA signaling, which activates SnRK2s (Sucrose non‐fermenting 1‐related protein kinase 2s) (Umezawa et al., 2009; Zhou et al., 2015). These SnRK2s then phosphorylate downstream transcription factors, including ABI3 (ABA INSENSITIVE 3) and ABI5, further regulating the expression of ABA‐responsive genes (Cai et al., 2017; Feng et al., 2014). Moreover, numerous studies indicate that various transcription factors regulate plant salt tolerance in a manner that is more or less dependent on ABA signal transduction pathways (Jin et al., 2023; Lee & Song, 2022; Li et al., 2022; Lin et al., 2020; Liu et al., 2007, 2025; Zhang et al., 2020).

Of particular note, during rice seed germination under high salt stress, the ABA signaling pathway is rapidly activated to regulate the gemination program. ABA significantly delays the germination process by inhibiting radicle elongation and regulating water balance and osmotic balance within the seed, thereby playing a critical protective role at the germination stage (Sajeev et al., 2024). Studies have shown that the ABA signaling response to salt stress during seed germination is a key determinant of germination success, and its intensity and duration directly influence the transition from germination to post‐germinative growth (Cutler et al., 2010; Han et al., 2019). Therefore, elucidating the molecular mechanism by which ABA signaling regulates rice seed germination under high salt stress is of great importance for understanding crop salt tolerance and for breeding salt‐tolerant rice varieties.

Protein ubiquitination requires the sequential catalysis of three types of enzymes: E1, E2, and E3 (Serrano et al., 2018). Current research has mostly focused on E3 ubiquitin ligases (Zhang et al., 2021, 2026; Zhang, Zhu, et al., 2024), while the specific functions of E2 ubiquitin‐conjugating enzymes remain poorly understood (Yang et al., 2017). Rice has 48 ubiquitin‐conjugating enzymes (Wu et al., 2025; Zhiguo et al., 2015), and only a few E2s have been functionally characterized (Li, Zhang, et al., 2023; Liu et al., 2021; Wang et al., 2023; Yu et al., 2024; Zhang, Wang, et al., 2024). Here, we found that two homologous rice ubiquitin‐conjugating enzyme genes, *OsUBC11* (LOC_Os01g62244) and *OsUBC12* (LOC_Os05g38550), are induced by ABA and various abiotic stresses. Phenotypic analyses showed that mutations in *OsUBC11/12* cause severe delays in seed germination under NaCl and ABA treatments. Further studies revealed that OsUBC11/12 interact with OsABI3, a core transcription factor in ABA signaling, to control its ubiquitination level. This study provides insights into the functions of E2‐conjugating enzymes in salt stress response, and the molecular mechanisms of ABA‐mediated seed germination.

## RESULTS

### *OsUBC11/12* are highly expressed in seeds and respond to multiple abiotic stresses

**Figure 1:**
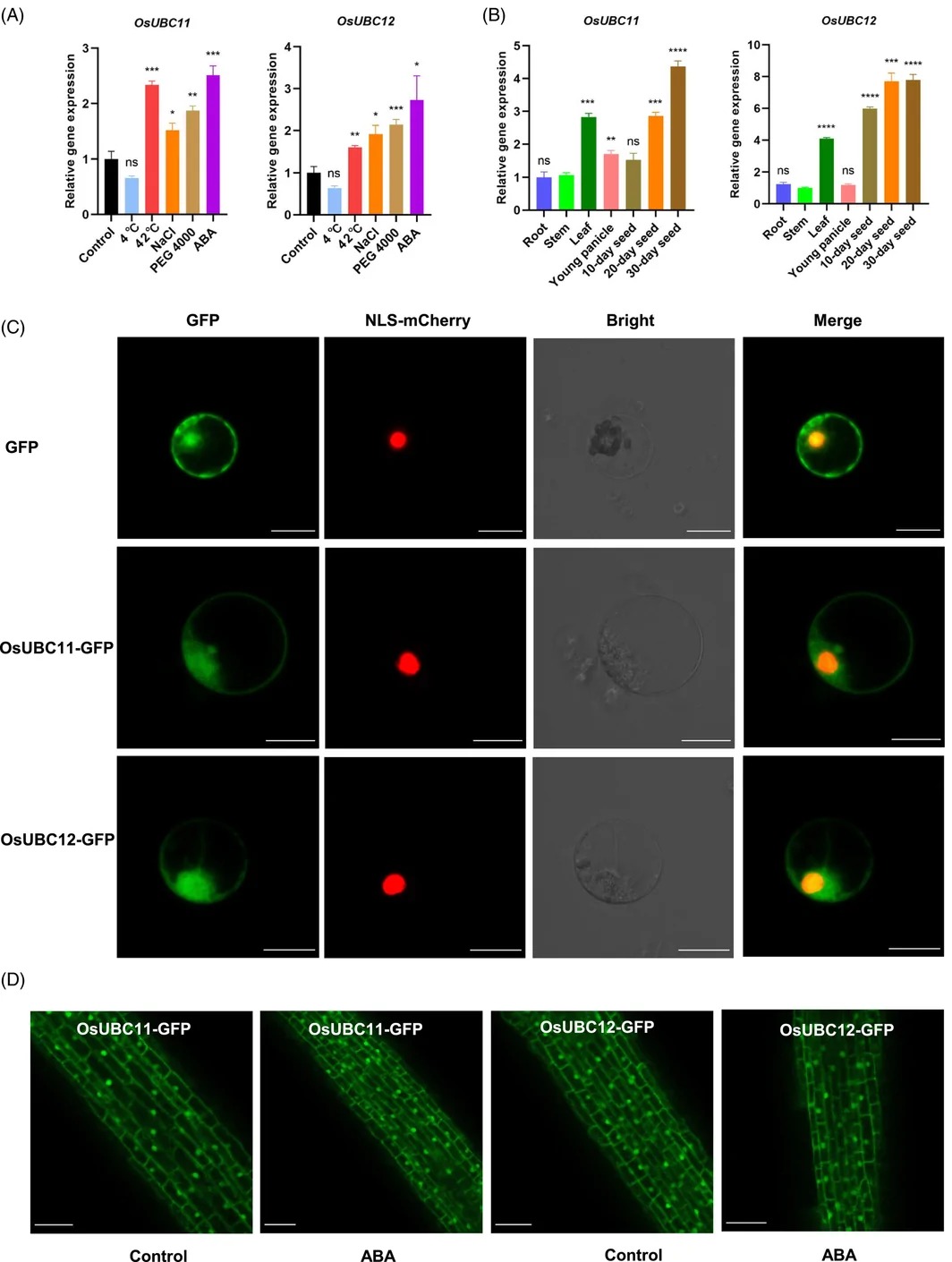
Multiple stress responsive *OsUBC11/12* genes encode predominantly nucleus‐localized proteins. (A) Stress responsive gene expression. Seven‐day‐old wild‐type rice seedlings (ZH11) grown at 29°C (control) were subjected to cold (4°C), heat (42°C), salt stress (100 mm NaCl), osmotic stress (20% PEG‐4000), and ABA (100 μm) treatment. (B) Tissue‐specific expression. Total RNA was extracted from various tissues of ZH11, and the expression level of *OsUBC11/12* was examined using RT‐qPCR. Data are presented as mean values ±SE, *n* = 3. Asterisks indicate significance levels when comparing to the control in *t*‐test (*P < 0.05; **P < 0.01; ***P < 0.001; ns, not significant at *P* < 0.05). (C, D) Subcellular localization. OsUBC11/12‐GFP transiently expressed in rice protoplasts with a nuclear marker (NLS‐mCherry) (C) or stably expressed in rice root cells (D) were observed. Bar = 20 μm in (C) and 50 μm in (D).

In our RNA‐seq data, we identified a class of key enzymes that catalyze protein ubiquitination, the E2 ubiquitin‐conjugating enzymes, which are extensively involved in the response of rice to abiotic stress. Among them, two homologous genes, *OsUBC11* and *OsUBC12*, belong to subfamily V of the rice ubiquitin‐conjugating enzyme family. To verify whether these two genes respond to abiotic stress, 7‐day‐old wild‐type (ZH11) rice seedlings were treated with water (negative control), cold (4°C), heat (42°C), salt (100 mm NaCl), osmotic stress (20% PEG 4000), or ABA (100 μm). RT‐qPCR analysis confirmed that the expression of *OsUBC11* and *OsUBC12* was significantly induced by NaCl, PEG, heat, and ABA (Figure 1A). To further investigate their function, we examined the tissue‐specific expression pattern of *OsUBC11* and *OsUBC12* by RT‐qPCR, which showed that both genes were expressed in all examined tissues, with particularly high expression in mature seeds (Figure 1B).

Subsequently, we examined the subcellular localization of OsUBC11 and OsUBC12 proteins using confocal microscopy. The results showed that OsUBC11/12‐GFP fusion proteins were localized both in the nucleus and cytosol of rice protoplasts, where most of the GFP signal co‐localized with the nuclear marker NLS‐mCherry (Figure 1C). Similar results were obtained in stably transformed rice roots with or without ABA treatment (Figure 1D). Together, these results demonstrate that OsUBC11/12 are transcriptionally activated by multiple abiotic stresses and ABA treatment, and their high expression in mature seeds suggests important roles during rice seed development and germination.

### OsUBC11/12 positively regulate rice seed germination under NaCl and ABA treatment

**Figure 2:**
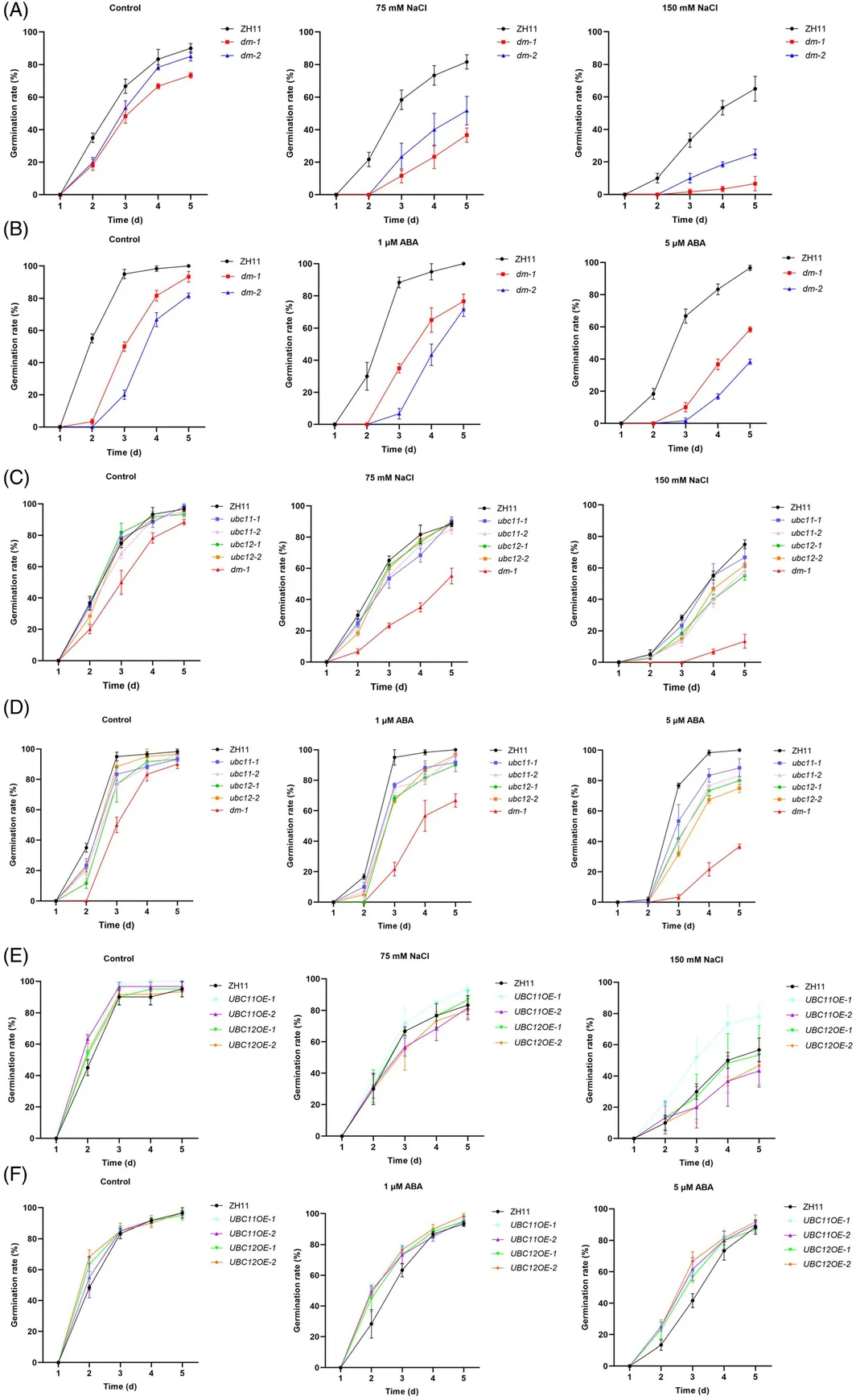
OsUBC11/12 positively regulates rice seed germination under NaCl and ABA treatment. Wild‐type ZH11, *OsUBC11/12* single and double (*dm‐1/‐2*) mutants (A–D), and overexpression lines (E, F) were germinated on ½‐MS agar medium containing water, NaCl or ABA and germination rates were monitored for 5 days. Data are presented as mean values ±SE, *n* = 3. In each biological replicate, 20 seeds were checked.

To investigate the function of *OsUBC11/12*, we generated *ubc11 ubc12* double loss‐of‐function mutants using the CRISPR‐Cas9 technology (Figure S1). Single mutants were obtained by crossing the double mutants with ZH11. Preliminary experiments showed that the loss of function of *OsUBC11/12* delayed seed germination and conferred hypersensitivity to NaCl and ABA during germination; moreover, during post‐germinative growth, the double mutants (*dm‐1/‐2*) were more sensitive to mannitol and ABA than ZH11 (Figure S2a,b). To further explore the function of *OsUBC11/12* in regulating rice seed germination, we performed germination assays on ½ MS medium supplemented with 0, 75 or 150 mm NaCl. On normal ½ MS medium, *ubc11 ubc12* double mutants germinated slightly more slowly than ZH11, whereas on NaCl‐containing medium the double mutants germinated significantly more slowly than ZH11 (Figure 2A). Similar experiments were performed on ½ MS medium supplemented with 0, 1 or 5 μm ABA. The results showed that on ABA‐containing medium, the double mutants also germinated significantly more slowly than ZH11 (Figure 2B). Single mutants showed an intermediate germination rate between ZH11 and the double mutant grown on NaCl‐ or ABA‐containing plates (Figure 2C,D), indicating functional redundancy in regulating germination between *OsUBC11* and *OsUBC12*.

We also generated overexpression lines of *OsUBC11* and *OsUBC12* driven by the constitutive *ubiquitin* promoter and confirmed them by Western blotting and RT‐qPCR (Figure S3). Germination assays on ½ MS medium containing NaCl or ABA showed that the overexpression lines of *OsUBC11* or *OsUBC12* germinated faster and were less sensitive to NaCl and ABA compared with ZH11 (Figure 2E,F), further supporting the positive regulatory role of *OsUBC11/12* in rice seed germination. However, these overexpression plants did not exhibit enhanced resistance to ABA, mannitol, and NaCl during growth following germination (Figure S4). Pre‐harvest sprouting (PHS), a phenomenon in which grains germinate on the mother plant directly before harvest, is a serious global problem for agricultural production, which is highly related to ABA responses (Tai et al., 2021). We checked the PHS in *OsUBC11/12* mutants and overexpression lines. The results showed that the loss‐of‐function mutants delayed PHS while overexpression lines were similar to ZH11 plants (Figure S5). Taken together, these results demonstrated that OsUBC11 and OsUBC12 are important for NaCl and ABA responses during rice seed germination.

### OsUBC11/12 negatively regulate the expression of NaCl‐ and ABA‐responsive genes

**Figure 3:**
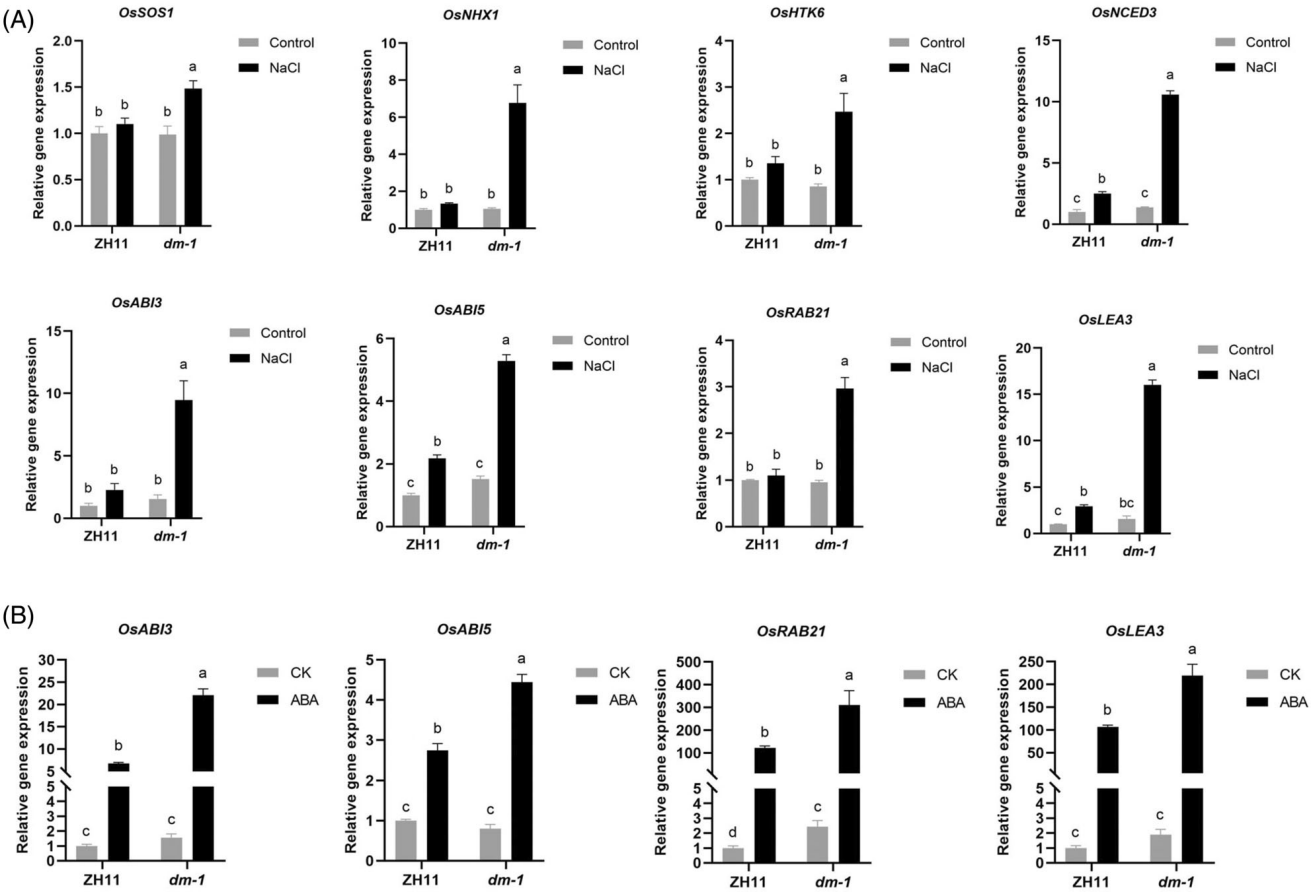
OsUBC11/12 negatively regulates salt stress and ABA‐responsive genes. Five‐day‐old seedlings of wild‐type ZH11 and *OsUBC11/12* double mutant (*dm‐1*) were transferred to an aqueous solution supplemented with or without NaCl (A) for 3 h or ABA (B) for 6 h, and total RNA was extracted for RT‐qPCR. Relative expression is the expression level of genes normalized to that of *Actin*. Error bars represent SE (*n* = 3). Different letters indicate significant differences for comparisons between two samples as determined by LSD test following anova analysis (*P* < 0.05).

To elucidate the underlying mechanism of OsUBC11/12 in NaCl and ABA responses, we performed RT‐qPCR analysis. The results showed that the NaCl‐induced up‐regulation of stress responsive genes such as *OsNHX1*, *OsHKT6*, *OsNCED3*, *OsABI3*, *OsABI5, OsRAB21*, and *OsLEA3* was significantly higher in the *ubc11 ubc12* double mutant than that in ZH11 (Figure 3A). Similarly, the ABA‐induced up‐regulation of downstream genes such as *OsABI3*, *OsABI5*, *OsRAB21*, and *OsLEA3* was also significantly higher in the double mutant than that in ZH11 (Figure 3B). Collectively, these results demonstrate that OsUBC11/12 negatively regulate the expression of NaCl‐ and ABA‐responsive genes.

### OsUBC11/12 interacts with OsABI3 and regulates its ubiquitination

To further dissect the mechanism by which OsUBC11/12 negatively regulate downstream gene expression, we sought to identify proteins in the ABA pathway that interact with OsUBC11/12. Because OsUBC11/12 predominantly regulate the expression of *OsABI3* and *OsABI5*, two key genes involved in ABA response and seed germination (Sano et al., 2016; Zou et al., 2007), and OsABI3 directly regulates the expression of *OsABI5* and *OsLEA3* (Guo et al., 2024; Zhang et al., 2023), we sought to dissect the relationship between OsUBC11/12 and OsABI3/5 in the later study. Firstly, we checked OsABI5 accumulation using a commercially available OsABI5 antibody in ZH11 and *ubc11 ubc12* double mutant with or without ABA treatment. The results showed that ABA treatment induced the accumulation of ABI5 protein as expected. In contrast, the accumulation level of OsABI5 was increased in the *ubc11 ubc12* mutant compared to that in ZH11 plants (Figure S6), suggesting that OsABI5 protein level is affected by OsUBC11/12 during seed germination.

**Figure 4:**
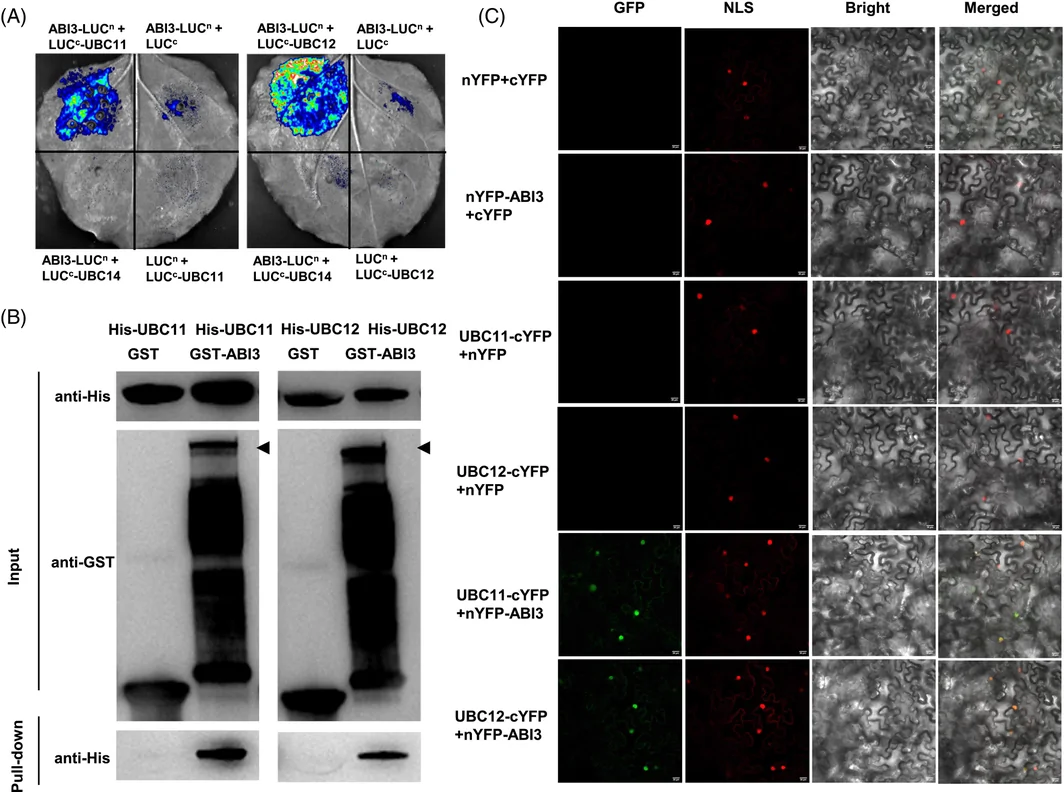
OsUBC11/12 interact with OsABI3 both *in vitro* and *in vivo*. (A) Split‐luciferase assays. OsABI3 and OsUBC11/12 were fused with the N‐terminal (nLUC) and C‐terminal (cLUC) portion of firefly luciferase, respectively. OsUBC14 was used as a negative control. Different combinations of constructs were agro‐infiltrated into tobacco leaves, and the chemiluminescence was observed. (B) Pull‐down assay. OsABI3 was fused with a GST tag, and OsUBC11/12 were fused with a His tag. After coincubation with both proteins, the proteins were immunoprecipitated with Glutathione‐Superflow resins and detected using an anti‐His antibody. GST was used as a negative control. Arrowhead points to GST‐ABI3. (C) split‐YFP assays. OsUBC11/12 and OsABI3 were fused with the C‐terminal (cYFP) and N‐terminal (nYFP) portion of YFP, respectively. Different combinations of constructs were agro‐infiltrated into tobacco leaves, and the fluorescence was observed. Bar = 20 μm.

We then checked the interaction between OsABI3 and OsUBC11/12. In split‐luciferase assays, both OsUBC11 and OsUBC12 interact with OsABI3 (Figure 4A). In pull‐down assays, GST‐tagged OsABI3 pulled down His‐tagged OsUBC11/12 (Figure 4B). The interaction was further confirmed by split‐YFP experiments in which the interaction was observed in the nucleus (Figure 4C). Together, our results demonstrate that OsUBC11/12 interact with OsABI3 both *in vitro* and *in vivo*.

**Figure 5:**
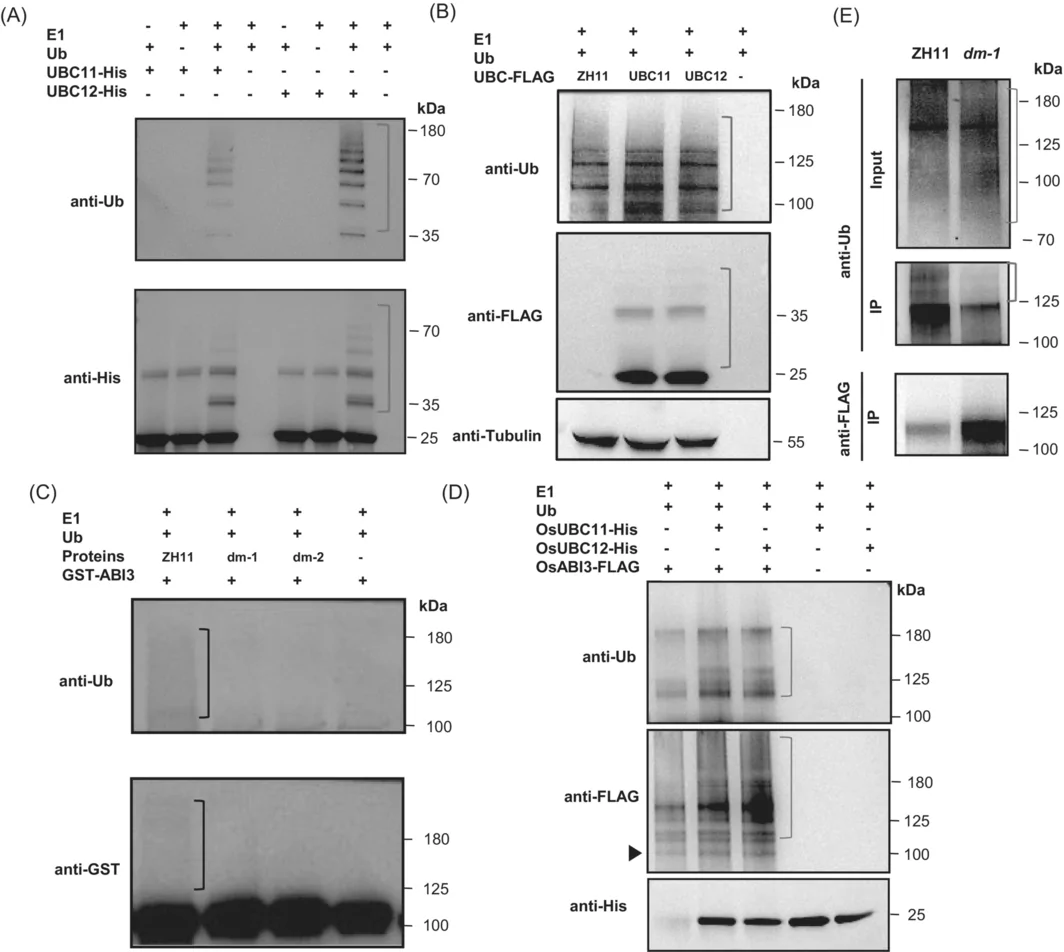
OsUBC11/12 control the ubiquitination level of OsABI3. (A, B) E2 ubiquitin conjugation assays. Recombinant OsUBC11/12‐His proteins (A) or immunoprecipitated OsUBC11/12‐FLAG proteins from transgenic seedlings (B) were incubated with ubiquitin (Ub) and E1, and checked with anti‐ubiquitin or anti‐His antibody (A). (C) Semi‐*in vitro* ubiquitination assays. Total active proteins were extracted from wild‐type ZH11 and *OsUBC11/12* double mutants (*dm‐1/‐2*) and incubated with purified GST‐ABI3 protein in the presence of Ub and E1. The ubiquitination of GST‐ABI3 was detected using anti‐Ub and anti‐GST antibodies. (D, E) Enhancement of OsABI3 ubiquitination by OsUBC11/12. Ub, E1, and purified UBC11/12‐His were incubated with ABI3‐FLAG protein immunoprecipitated from transgenic plants (D). ABI3‐FLAG overexpression proteins from ZH11 and *dm‐1* were immunoprecipitated with anti‐FLAG from ABA‐treated plants, and the ubiquitination levels of ABI3‐FLAG proteins were assessed using anti‐Ub and anti‐FLAG antibodies (E). Brackets and arrowhead point to the ubiquitinated and non‐ubiquitinated bands, respectively.

OsUBC11/12 encode E2 ubiquitin‐conjugating enzymes (Han et al., 2023), therefore, we performed *in vitro* E2 ubiquitin conjugation assays. The results showed that OsUBC11/12‐His proteins conjugate multiple ubiquitin molecules in the presence of Ub and E1, resulting in a large mobility shift (Figure 5A; Figure S7), indicating that UBC11/12 possess ubiquitin‐conjugating enzyme activity. Indeed, immunoprecipitated OsUBC11/12‐FLAG proteins from rice seedlings were ubiquitinated (Figure 5B). To further confirm whether OsUBC11/12 are involved in ubiquitinating OsABI3, we performed semi‐*in vivo* ubiquitination assays. Total active proteins were extracted from ZH11 and *ubc11 ubc12* double mutant and then incubated with purified GST‐OsABI3. The results showed that the ubiquitination level of GST‐OsABI3 was reduced in the *ubc11 ubc12* mutant when compared to that in ZH11 (Figure 5C). We further demonstrated that *in vitro*‐purified OsUBC11/12‐His protein could also enhance the ubiquitination level of OsABI3‐FLAG immunoprecipitated from rice seedlings (Figure 5D). Finally, we compared the ubiquitination levels of OsABI3‐FLAG protein in ZH11 versus *ubc11 ubc12* mutant background. The results showed that although OsABI3‐FLAG protein levels were much higher in the double‐mutant background than in ZH11 background, the ubiquitination level of OsABI3‐FLAG was higher in ZH11 background than that in the double‐mutant background (Figure 5E; Figure S8), indicating that the loss of function of *OsUBC11/12* reduces the ubiquitination of OsABI3‐FLAG *in vivo*. Collectively, these results demonstrate that OsUBC11/12 ubiquitinate OsABI3.

### OsUBC11/12 regulates OsABI3 stability

**Figure 6:**
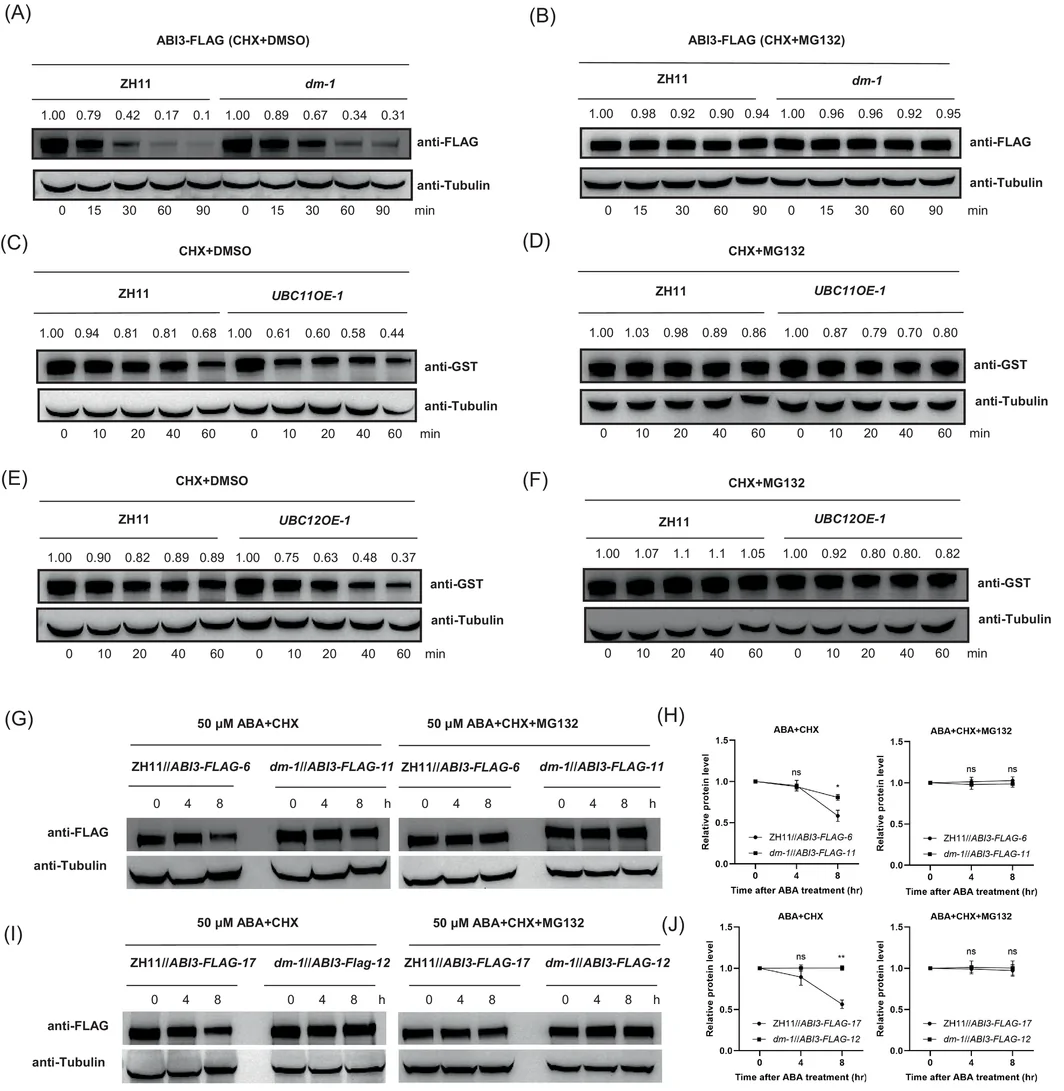
OsUBC11/12 regulate OsABI3 protein stability. (A–F) Regulation of OsABI3 protein stability in cell‐free degradation assays. Total proteins extracted from *ABI3‐FLAG* overexpression lines in the wild‐type ZH11 background or in the *OsUBC11/12* double‐mutant (*dm‐1*) background were incubated with or without MG132 over the indicated time course, and the level of OsABI3 was detected using anti‐FLAG antibody (A, B). The purified recombinant GST‐ABI3 protein was incubated with the active protein extracts from ZH11 and *OsUBC11/12* overexpression lines (*UBC11/12OE‐1*), in the presence or absence of MG132, and the level of OsABI3 was detected using anti‐GST antibody (C–F). Tubulin was used as a loading control, and CHX was used to inhibit protein synthesis. The relative protein intensity normalized to the level of Tubulin is shown above the gels. (G–J) Regulation of OsABI3 protein accumulation in plants. Os*ABI3‐FLAG* overexpression lines in ZH11 or in *dm‐1* mutant background were treated with ABA for 0, 4, and 8 h, and total proteins were extracted for Western blotting analysis. MG132 was used to block 26S proteasome‐mediated protein degradation, while CHX was used to inhibit protein synthesis. Tubulin was used as a loading control, and the protein levels were quantified using ImageJ software. Error bars represent SE (*n* = 3). *P < 0.05; **P < 0.01; ns, not significant at *P* < 0.05 in *t*‐test.

To further explore the biological significance of OsABI3 ubiquitination, we co‐expressed *OsUBC11/12* and *OsABI3* in *N. benthamiana*. The results showed that OsABI3 protein levels were reduced when *OsUBC11/12* was co‐expressed (Figure S9), suggesting that OsUBC11/12‐mediated ubiquitination of OsABI3 promotes its degradation. We then overexpressed *OsABI3‐FLAG* in ZH11 and *ubc11 ubc12* double‐mutant background driven by the constitutive ubiquitin promoter and performed cell‐free degradation assays. The results showed that OsABI3‐FLAG degraded more slowly in the double‐mutant background than that in ZH11 background (Figure 6A). Addition of MG132 abolished this difference (Figure 6B), indicating that the loss‐of‐function of OsUBC11/12 slows the degradation of OsABI3‐FLAG. Next, we extracted active proteins from ZH11 and *OsUBC11/12* overexpression lines, and co‐incubated with *in vitro*‐purified GST‐OsABI3 to examine the degradation rate of GST‐OsABI3. The results showed that GST‐OsABI3 degraded faster in the presence of total protein from *OsUBC11/12* overexpression lines than that from ZH11 (Figure 6C,E). Again, addition of MG132 attenuated this difference (Figure 6D,F), indicating that overexpression of OsUBC11/12 significantly accelerates the degradation of OsABI3. Together, these results demonstrate that OsUBC11/12 regulate OsABI3 stability *in vitro*.

To investigate whether OsUBC11/12 regulate OsABI3 stability *in vivo*, we selected *OsABI3‐FLAG* overexpression lines in ZH11 and *ubc11 ubc12* double‐mutant backgrounds with comparable transgenic expression (Figure S8). These materials were treated with ABA for 0, 4, or 8 h, and OsABI3‐FLAG protein levels were examined. The results showed that upon ABA treatment, the OsABI3‐FLAG protein level decreased more slowly in the double‐mutant background than that in the ZH11 background, and the addition of MG132 suppressed the degradation of OsABI3‐FLAG in both backgrounds (Figure 6G–J). These results indicate that OsUBC11/12 regulate OsABI3 stability *in vivo*. We also checked the protein accumulation level of OsUBC11/12‐FLAG under ABA and NaCl treatment. The results showed that neither ABA nor NaCl treatment affected the accumulation of OsUBC11/12 (Figure S10). Taken together, these results demonstrate that OsUBC11/12 regulate OsABI3 stability under ABA treatment.

Delayed Seed Germination 1 (OsDSG1) is a functional E3 ubiquitin ligase that regulates OsABI3 stability (Park et al., 2010). The interaction between OsUBC11/12 and OsDSG1 was confirmed in yeast two‐hybrid assays, pull‐down assays, and split‐luciferase assays (Figure S11). These results indicate that OsUBC11/12 and OsDSG1 may function together to regulate OsABI3 ubiquitination. To check whether OsDSG1 also functions in regulating rice seed germination, we generated OsDSG1‐edited lines (Figure S12) and examined seed germination phenotypes on NaCl‐ and ABA‐containing media. The results showed that on normal ½ MS medium, *dsg1* mutants germinated at a rate similar to wild‐type, but on medium supplemented with NaCl or ABA, *dsg1* mutants germinated more slowly than ZH11 (Figure S13a,b), indicating that OsDSG1 also negatively regulates rice seed germination. As expected, *dsg1* mutants delayed PHS (Figure S14a,b). Collectively, these results suggest that OsDSG1 may be involved in OsABI3 ubiquitination.

### The function of OsABI3 in seed germination is partially dependent on OsUBC11/12

**Figure 7:**
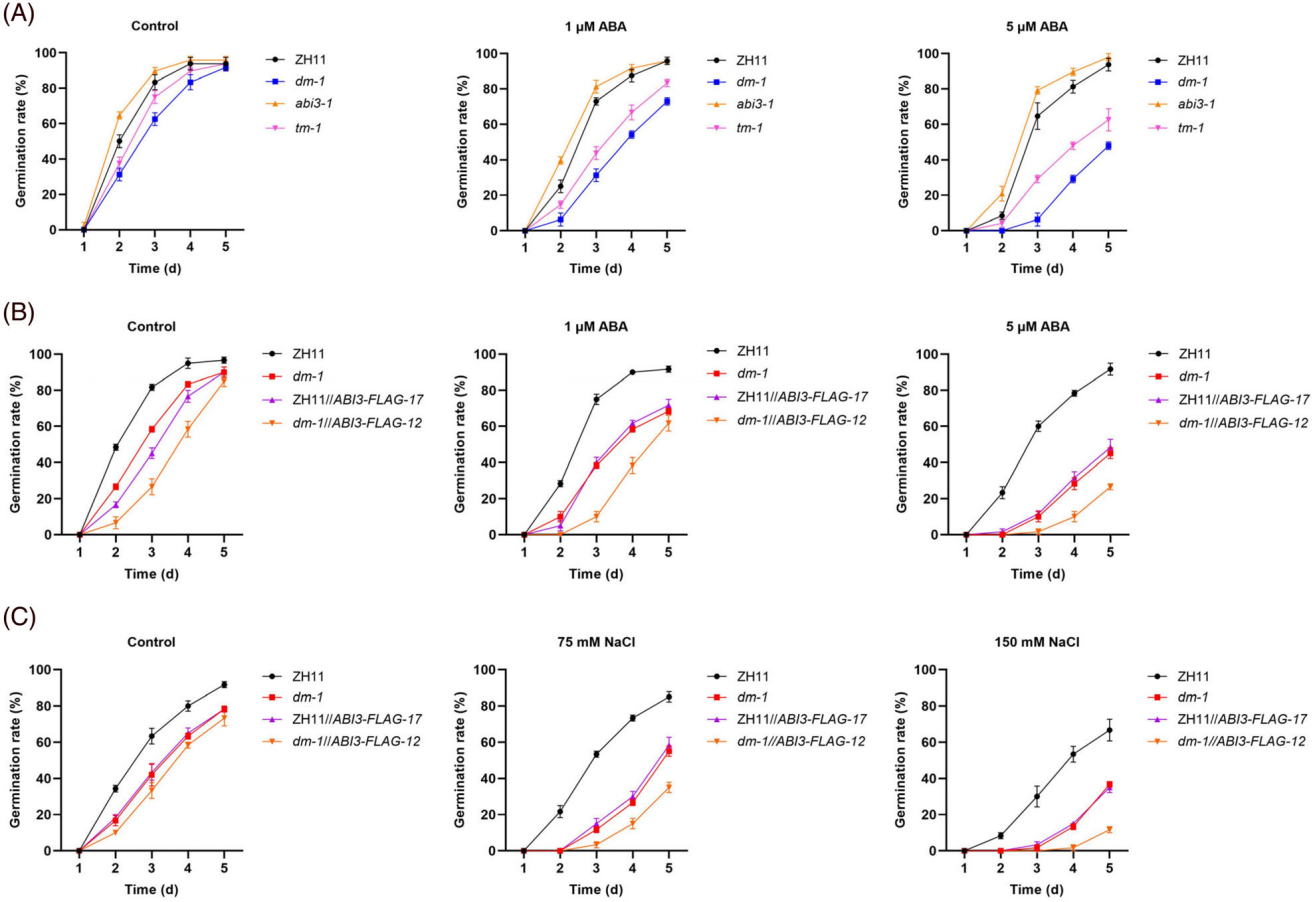
The function of OsABI3 in seed germination is partially dependent on OsUBC11/12. (A) Genetic analysis. Wild‐type ZH11, *ubc11 ubc12* double mutant (*dm‐1*), *abi3* mutant, and *ubc11 ubc12 abi3* triple mutant (*tm‐1*) were germinated on ½‐MS agar medium containing different concentrations of ABA and their germination was checked for 5 days. (B, C) Dependence of OsABI3 on OsUBC11/12. ZH11, *dm‐1*, *OsABI3* overexpression line in ZH11 background (ZH11//ABI3‐FLAG), and in the *dm‐1* background (*dm‐1*//ABI3‐FLAG) were germinated on ½‐MS agar medium containing different concentrations of ABA (B) or NaCl (C), and their germination was checked for 5 days. Error bars represent SE (*n* = 3).

To investigate the genetic relationship between *OsUBC11/12* and *OsABI3*, we generated *abi3* single mutant and *ubc11 ubc12 abi3* triple mutant, and examined seed germination phenotypes on ABA‐containing medium. The results showed that *abi3* single mutant germinated faster while *ubc11 ubc12* double mutant germinated slower than ZH11, whereas *ubc11 ubc12 abi3* triple mutant germinated at an intermediate rate between ZH11 and *ubc11 ubc12* double mutant (Figure 7A). We also overexpressed OsABI3 in ZH11 and *ubc11 ubc12* double‐mutant background, respectively. The results showed that the germination phenotype of *OsABI3* overexpression lines was similar to that of the *ubc11 ubc12* double mutant, whereas the *OsABI3* overexpression line in the double‐mutant background showed a more sensitive phenotype to NaCl and ABA (Figure 7B,C). Together, these results demonstrate that the function of OsABI3 in seed germination is partially dependent on OsUBC11/12.

## DISCUSSION

**Figure 8:**
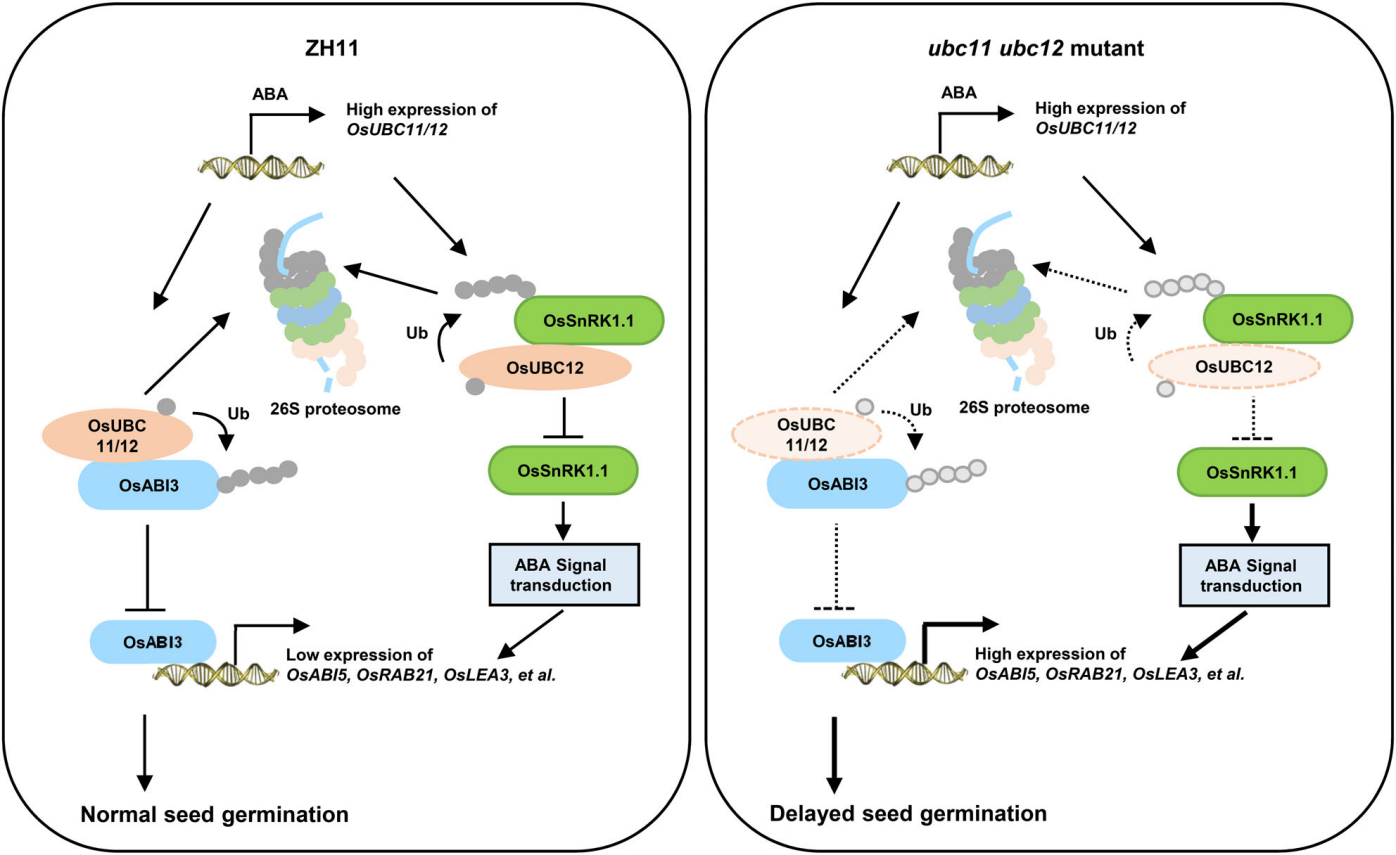
A working model for the role of OsUBC11/12 in ABA‐mediated seed germination in rice. In the wild‐type ZH11, ABA and abiotic stresses such as salt stress up‐regulate the expression of *OsUBC11/12*. OsABI3 directly induces the expression of ABA‐responsive genes such as *OsABI5*, *OsRAB21*, and *OsLEA3*. OsUBC11/12 interact with OsABI3 to regulate its ubiquitination level, leading to OsABI3 degradation, which results in reduced expression of ABA‐responsive genes and enhanced seed germination. Additionally, OsUBC12 interacts with OsSnRK1.1 to promote its ubiquitination, thereby inhibiting the expression of ABA‐responsive genes and further promoting seed germination. In the *ubc11 ubc12* double mutant, both OsABI3 and OsSnRK1.1 accumulate, leading to increased expression of ABA‐responsive genes and consequently delayed seed germination.

E2 ubiquitin‐conjugating enzymes are key components of the ubiquitin‐proteasome system, playing critical roles in plant responses to various abiotic stresses, including drought, salinity, cold, and heat (Liu et al., 2020; Xu & Xue, 2019). In this study, we identified two rice ubiquitin‐conjugating enzymes, OsUBC11 and OsUBC12, as positive regulators of seed germination under abiotic stress conditions, particularly during NaCl and ABA treatment. Our findings reveal a novel mechanism by which OsUBC11/12 promote seed germination by directly interacting with and ubiquitinating OsABI3, a key transcription factor in the ABA signaling pathway, thereby reducing its stability and attenuating ABA‐responsive gene expression (Figure 8).

The expression patterns of *OsUBC11* and *OsUBC12* suggest their important roles in stress response and seed germination. Both genes were significantly induced by NaCl, PEG 4000, and ABA, consistent with their functions in abiotic stress adaptation (Figure 1A). Similar observations have been reported for other plant UBC genes. For instance, a systematic analysis of the E2 ubiquitin‐conjugating enzyme family identified seven UBC genes (OsUBC4/6/7/12/25/27/48) that regulate seed germination, with OsUBC27 and OsUBC48 acting through the ABA pathway (Wu et al., 2025). The predominant nuclear localization of OsUBC11/12 (Figure 1C,D) supports their involvement in regulating transcription factors or nuclear proteins, such as OsABI3. Indeed, the interaction between OsUBC11/12 and OsABI3 was observed in the nucleus in tobacco leaves (Figure 4C). Notably, the high expression levels in mature seeds (Figure 1B) point to a specific role during seed development and germination, a phase tightly controlled by ABA and environmental cues. Phenotypic analyses clearly demonstrated that loss of function of *OsUBC11/12* delays seed germination, especially under NaCl or ABA treatment, while overexpression accelerates germination and reduces stress sensitivity (Figure 2). These results establish OsUBC11/12 as positive regulators of germination under stress. The intermediate phenotype of single mutants (Figure 2C,D) indicates functional redundancy between OsUBC11 and OsUBC12, a common feature among E2 enzyme families in plants. Interestingly, a similar pattern of functional redundancy has been reported for Arabidopsis UBC32, UBC33, and UBC34, where single mutants exhibited germination rates comparable to wild‐type under salt stress or ABA treatment, but double mutants showed significantly greater reduction in germination rate (Wang et al., 2025). This suggests that functional redundancy among closely related E2 family members may be a conserved mechanism in plant stress responses.

In agricultural production, we often prefer rice seeds that germinate more readily, which improves sowing efficiency. However, faster‐germinating seeds are often more prone to pre‐harvest sprouting (PHS) under continuous rainy weather, leading to severe yield losses (Yang et al., 2025). *Seed Dormancy 6* (*SD6*), encoding a basic helix–loop–helix (bHLH) transcription factor, and another bHLH factor inducer of C‐repeat binding factors expression 2 (ICE2) function antagonistically in controlling seed dormancy by directly regulating the ABA catabolism gene *ABA8OX3* (Xu et al., 2022). Interestingly, genome editing of *SD6* and its wheat homologs alleviates PHS in cereals under field conditions (Xu et al., 2022). We observed that the *ubc11 ubc12* double mutants have a lower PHS rate than ZH11 (Figure S5). Whether manipulating these genes has agricultural application potential needs to be tested under field conditions in the future.

In the current study, we demonstrated that OsUBC11/12 interact with OsABI3 and regulate OsABI3 stability (Figures 4, 5, 6). Genetic experiments showed that OsABI3 is not the sole target of OsUBC11/12 (Figure 7A). Notably, a recent study showed that OsUBC12 enhances cold tolerance during japonica rice germination by promoting the proteasomal degradation of OsSnRK1.1 (Zhang, Wang, et al., 2024), which belongs to the energy sensor family to balance the developmental growth and stress survival (Broeckx et al., 2016). In Arabidopsis, SnRK1.1 plays a central role in sugar and ABA signaling, and its overexpression delays germination and growth (Jossier et al., 2009). Therefore, OsSnRK1.1 might be an additional targeting protein for ABA‐mediated seed germination under salt stress conditions (Figure 8).

Post‐translational regulation of ABA signaling components via ubiquitination has been extensively studied (Yu et al., 2016). In Arabidopsis, the E3 ubiquitin ligase AIP2 (ABI3‐interacting protein 2) targets ABI3 for degradation, negatively regulating ABA signaling during seed germination (Zhang et al., 2005). Similarly, a U‐box E3 ubiquitin ligase, PLANT U‐BOX 8 (PUB8), physically interacts with ABI3 and ABI5 and negatively regulates ABA responses during early Arabidopsis seedling growth (Li, Li, et al., 2023). In the current study, we observed that OsUBC11/12 interact with OsDSG1 (Figure S11), suggesting that OsUBC11/12 may serve as the cognate E2 conjugating enzyme for OsDSG1‐mediated OsABI3 ubiquitination. Furthermore, our observation that *dsg1* mutants exhibit delayed germination under NaCl and ABA treatment (Figure S13), phenocopying the *ubc11 ubc12* double mutant, further supports a functional relationship among these genes. However, whether OsDSG1 is the primary E3 ligase working with OsUBC11/12, or whether additional E3 ligases are also involved, remains to be determined. Future studies using co‐immunoprecipitation coupled with mass spectrometry may help identify the specific E3 ligase(s) that work together with OsUBC11/12.

In summary, we revealed a new molecular mechanism underlying the function of OsUBC11/12 in rice seed germination, in which OsUBC11/12 interact with and ubiquitinate OsABI3, leading to its proteasomal degradation. This reduces OsABI3 protein levels, attenuates ABA‐responsive gene expression, and ultimately promotes seed germination under stress conditions.

## MATERIALS AND METHODS

### Plant materials and genetic constructs

All rice materials used in this study were in the japonica variety ZH11 background. To generate gene‐edited mutants of *OsUBC11*, *OsUBC12*, *OsDSG1*, and *OsABI3*, we designed specific sgRNAs for each gene and cloned them into the CRISPR‐Cas9 system. For overexpression of *OsUBC11*, *OsUBC12*, and *OsABI3*, the full‐length coding sequences (CDSs) of these genes were cloned into the pCAMBIA 1300‐FLAG vector. All constructs were verified by sequencing and introduced into rice via *Agrobacterium*‐mediated stable transformation. To obtain single mutants of *OsUBC11* and *OsUBC12*, the double mutant was crossed with wild‐type ZH11 plants, and homozygous single mutants were isolated from the progeny. Additionally, we generated triple mutants and double‐mutant backgrounds overexpressing *OsABI3* by introducing *OsABI3* mutations or overexpression constructs into the *OsUBC11 OsUBC12* double mutant using the same approach. All primers used are listed in Table S1.

### Growth conditions and phenotypic analyses

For seedling‐based experiments, rice seeds were soaked in water at 37°C for 2 days, then transferred onto filter paper to germinate. Subsequently, seedlings were transferred to 96‐well plates floating on Kimura B hydroponic solution and grown in a growth chamber under the following conditions: 20000 lux white light (12‐h light/12‐h dark cycle), 29°C, and 65% relative humidity. For seed germination assays, healthy, plump seeds were dehulled and surface‐sterilized with 75% ethanol for 5 min, followed by 20% sodium hypochlorite for an additional 20 min. After sterilization, seeds were rinsed thoroughly with sterile water, air‐dried on sterile filter paper, and then placed on 1/2 MS plates containing different concentrations of NaCl or ABA. Plates were placed horizontally in a plant growth chamber. For stress treatment assays on 1/2 MS plates, rice seeds were first germinated on 1/2 MS plates as described above. Uniformly germinated seeds were then transferred to 1/2 MS plates supplemented with ABA, mannitol, or NaCl for further growth. Plates were placed at a tilted angle in the plant growth chamber. For pre‐harvest sprouting assays, mature panicles at the same developmental stage were harvested, placed in water, and incubated at 30°C for 7 days with daily water changes. After 7 days, grains with a germinated embryo longer than half the grain length were counted as sprouted, and the germination rate was calculated.

### Subcellular localization

To determine the subcellular localization of OsUBC11 and OsUBC12, their CDSs were amplified and cloned into the pCAMBIA 1300‐GFP vector. The verified constructs were co‐expressed with a nuclear marker (NLS‐mCherry) in rice protoplasts or in stably transformed rice plants. Fluorescence signals were observed under a confocal microscope (Zeiss LSM A710). All primers are listed in Table S1.

### Yeast two‐hybrid assay

The full‐length CDSs of *OsUBC11* and *OsUBC12*, as well as the CDS of *OsDSG1*, were cloned into pGADT7 or pGBKT7 vectors to generate bait and prey constructs, respectively. Different combinations were co‐transformed into the yeast strain Y2HGold using a commercial kit and grown on selective media at 30°C for 2–4 days. All primers are listed in Table S1.

### Pull‐down assay

The full‐length CDSs of *OsUBC11*, *OsUBC12*, *OsDSG1*, and *OsABI3* were cloned into pET28a and pGEX4T‐1 vectors to generate His‐OsUBC11, His‐OsUBC12, GST‐OsDSG1, and GST‐OsABI3 fusion proteins, respectively. The constructs were transformed into *E. coli* BL21 competent cells, and positive clones were induced with 300 μm isopropyl‐β‐D‐1‐thiogalactopyranoside (IPTG) at 16°C overnight. Fusion proteins with His, MBP, or GST tags were purified using Ni‐NTA beads, amylose agarose beads, or glutathione‐Superflow resin, respectively. For pull‐down assays, different combinations of recombinant proteins were incubated in pull‐down buffer [20 mm Tris–HCl (pH 7.4), 200 mm NaCl, and 1 mm EDTA] with rotation for 2 h. Beads were then washed 3–5 times with pull‐down buffer and boiled in 2× SDS loading buffer. Samples were resolved by SDS‐PAGE and analyzed using anti‐His, anti‐MBP, or anti‐GST antibodies. All primers are listed in Table S1.

### Split‐luciferase and split‐YFP assays

The full‐length CDSs of *OsUBC11*, *OsUBC12*, *OsDSG1*, and *OsABI3* were amplified and fused with nLUC or cLUC to generate cLUC‐UBC11, cLUC‐UBC12, DSG1‐nLUC, and ABI3‐nLUC constructs, or fused with cYFP or YFP to generate UBC11‐cYFP, UBC12‐cYFP, and nYFP‐ABI3 constructs. Different combinations were transformed into *Agrobacterium tumefaciens* strain GV3101 and infiltrated into *N. benthamiana* leaves together with P19. Luciferase luminescence was detected using a Tanon 5200 image analyzer, and YFP fluorescence signals were observed under a confocal microscope (Zeiss LSM 710). All primers are listed in Table S1.

### Ubiquitin‐conjugating enzyme activity assay

For *in vitro* assay, the full‐length CDSs of *OsUBC11* and *OsUBC12* were cloned into the pET28a vector to generate UBC11‐His and UBC12‐His fusion proteins. His‐E1, UBC11‐His, UBC12‐His, and His‐ubiquitin (His‐Ub) proteins were purified using Ni‐NTA beads. The mutated form of UBC11/12 (C92A) at the catalytic site was prepared by overlapping PCR. Different combinations were incubated in reaction buffer containing 50 mm Tris–HCl (pH 7.5), 20 mm ZnCl_2_, 5 mm MgCl_2_, 2 mm ATP, 2 mm DTT, and 50 μm MG132 on a shaker at 30°C. After 1.5 h of incubation, the reactions were terminated by adding SDS loading buffer. The E2 ubiquitin‐conjugating activities of UBC11 and UBC12 were detected using anti‐ubiquitin and anti‐His antibodies. For semi‐*in vitro* assay, OsUBC11/12‐FLAG were immunoprecipitated from *OsUBC11/12* overexpression lines. The E2 ubiquitin‐conjugating activities of UBC11 and UBC12 were detected using anti‐ubiquitin and anti‐FLAG antibodies; anti‐Tubulin was used as a loading control.

### Semi‐*in vitro* and *in vivo* ubiquitination assays

His‐Ub, His‐E1, His‐E2, UBC11/12‐His, and GST‐ABI3 proteins were purified. Active protein extracts from ZH11 and the *OsUBC11/12* double mutants were used. OsABI3‐FLAG was immunoprecipitated from *OsABI3‐FLAG* overexpression lines. Different combinations were incubated in reaction buffer containing 50 mm Tris–HCl (pH 7.5), 20 mm ZnCl_2_, 5 mm MgCl_2_, 2 mm ATP, 2 mm DTT, and 50 μm MG132 on a shaker at 30°C. After 2 h of incubation, the reactions were terminated by adding SDS loading buffer. Ubiquitination of GST‐ABI3 was detected using anti‐ubiquitin, anti‐GST, and anti‐FLAG antibodies. For *in vivo* assay, ABI3‐FLAG protein was immunoprecipitated from overexpression lines in the wild‐type ZH11 or *OsUBC11/12* double‐mutant background using FLAG‐conjugated resin. After adding SDS loading buffer and SDS‐PAGE, the ubiquitination level of ABI3‐FLAG was detected using an anti‐ubiquitin antibody.

### Cell‐free degradation assay

To assess the degradation rate of ABI3‐FLAG protein in ZH11 and *OsUBC11/12* double‐mutant backgrounds, total protein was extracted from rice transgenic seedlings using extraction buffer [50 mm Tris‐MES (pH 8.0), 0.5 m sucrose, 1 mm MgCl_2_, 10 mm EDTA, and 5 mm DTT] freshly supplemented with protease inhibitor cocktail (cOmplete Mini tablets). The protein mixture was then incubated with 10 mm ATP and 50 μm cycloheximide (CHX) at 29°C for 0 to 90 min, with or without 200 μm MG132. After SDS‐PAGE, the abundance of ABI3‐FLAG was detected by western blotting. To assess the degradation rate of ABI3 protein when incubated with active protein extracts from ZH11 and *OsUBC11/12* overexpression lines, total protein was extracted from these lines using the same extraction buffer and then co‐incubated with purified GST‐ABI3 protein; GST‐ABI3 abundance was detected by Western blotting.

### *In vivo* protein stability analysis

To analyze ABI3‐FLAG protein stability in ZH11 and *OsUBC11/12* double‐mutant rice plants, we generated ABI3‐FLAG overexpression lines in ZH11 and *OsUBC11/12* double‐mutant backgrounds. Gene expression levels and ABI3‐FLAG protein abundance were first examined, and lines with comparable expression levels were selected for further study. These plants were treated with ABA, and samples were collected at different time points. The abundance of ABI3‐FLAG protein was then detected by Western blotting. Three independent experiments were performed, and each immunoblot was quantified using ImageJ software. To examine the stability of ABI3‐FLAG protein expressed in *N. benthamiana*, OsUBC11‐GFP, OsUBC12‐GFP, or GFP was co‐expressed with ABI3‐FLAG in *N. benthamiana* leaves. After 3 days, samples were collected and total protein was extracted. Protein samples were analyzed by Western blotting.

### RT‐qPCR analysis

For analysis of downstream marker gene expression in response to ABA or NaCl, RNA was extracted from rice seedlings germinated with or without ABA or NaCl treatment. For RT‐qPCR, total RNA was extracted using the RNA Prep Pure Plant Kit (Tiangen) and reverse‐transcribed using 5× PrimeScript RT Master Mix (Takara) with oligo(dT) primers (Takara). RT‐qPCR was performed using the SuperReal PreMix Plus Kit (Tiangen) on a CFX96 real‐time system (Bio‐Rad). Relative expression was calculated as the expression of the target gene normalized to *ACTIN*. All primers are listed in Table S1.

## AUTHOR CONTRIBUTIONS

J‐XL and T‐CX designed the experiments; T‐CX, TQ, CY, and J‐MW performed the experiments; J‐XL, T‐CX, and H‐PL analyzed the data; J‐XL, T‐CX, and H‐PL wrote the paper.

## CONFLICT OF INTEREST

The authors declare no competing interests.

## Supporting information


**Figure S1.** Characterization of the *OsUBC11/12* single and double mutants.
**Figure S2.** Regulation of seed germination and abiotic stress responses by OsUBC11/12.
**Figure S3.** Validation of the transgene expression.
**Figure S4.** Regulation of seedling growth by OsUBC11/12.
**Figure S5.** Regulation of pre‐harvest sprouting by OsUBC11/12.
**Figure S6.** Regulation of OsABI5 by OsUBC11/12.
**Figure S7.** Auto‐ubiquitination activity of OsUBC11/12.
**Figure S8.** Validation of transgene expression.
**Figure S9.** Regulation of OsABI3 accumulation by OsUBC11/12.
**Figure S10.** Accumulation of OsUBC11/12 proteins in response to ABA and NaCl treatment.
**Figure S11.** Interaction between OsUBC11/12 and OsDSG1.
**Figure S12.** Characterization of the *OsDSG1* single mutants.
**Figure S13.** Phenotypic analysis of the *dsg1* mutants.
**Figure S14.** Regulation of pre‐harvest sprouting by OsDSG1.

**Table S1.** Primers used in this study.

## Data Availability

All data supporting the findings of this study are available within the paper and its Supplementary Information.
