# Supplementary material for: The E2 ubiquitin‐conjugating enzymes OsUBC11 and OsUBC12 promote rice seed germination by ubiquitinating and destabilizing OsABI3 in the ABA signaling pathway
Source: Plant J. 2026 Sep 10;127(5):e71096. doi: 10.1111/tpj.71096 (PMC13559216; doi:10.1111/tpj.71096)
Supplement: Supplementary file 1 [file TPJ-127-0-s001.pdf]

Table S1. Primers used in this study.

Name	Forward primer (5'-3')	Reverse primer (5'-3')	Purpose
sgUBC11	GAGGTAAATCTGACAGTTGGGTTTTAGAGCTAGAAATAGCAAGTTA	CCAACTGTCAGATTTACCTCAACCTGAGCCTCAGCGCAGC	Gene editing
sgUBC12	GAGGAGGCTCGCCTGGCTCGGTTTTAGAGCTAGAAATAGCAAGTTA	CGAGCCAGGCGAGCCTCCTCAACCTGAGCCTCAGCGCAGC	Gene editing
sgDSG1	GCTTCTCTGTACCTAAGAAGGTTTTAGAGCTAGAAATAGCAAGTTA	CTTCTTAGGTACAGAGAAGCAACCTGAGCCTCAGCGCAGC	Gene editing
sgABI3	GAAGTCCGGGAGCAACGGGAGTTTTAGAGCTAGAAATAGCAAGTTA	TCCCGTTGCTCCCGGACTTCAACCTGAGCCTCAGCGCAGC	Gene editing
UBC11-JD	CTGGTGTCCAACGCTCACTC	AGCCCTTTTCATGTGCTTGC	Sequencing
UBC12-JD	CACGCCTCCACAAATAAAGC	ATGATGGTGACCTGCCACTC	Sequencing
DSG1-JD	ACCACACCCCTACAATCAGTT	TCACAGGTTGCCTCCAGAATA	Sequencing
ABI3-JD	CTCGCCTACATCTCGCGCTCTC	GCCTCCATCACCGCCTTCGTAT	Sequencing
1301-UBC11-FLAG	ACGGGGTACCATGGCGGCTACCATCAGC	CGGACTAGTGAGCATTTCTTGTGATTTCCTTAC	Overexpression
1301-UBC12-FLAG	ACGGGGTACCATGGCGACTGCCGCGAGCCA	CGGACTAGTGAGCATTTCCTGTGATTTACGAAC	Overexpression
1301-ABI3-FLAG	CGCGGATCCATGGACGCCTCCGCCGGCT	GGACTAGTGATGCTAACCGCCATCTGGCTCATGG	Overexpression
1301-UBC11-GFP	ACGGGGTACCATGGCGGCTACCATCAGC	CGGACTAGTGAGCATTTCTTGTGATTTCCTTAC	Subcellular localization
1301-UBC12-GFP	ACGGGGTACCATGGCGACTGCCGCGAGCCA	CGGACTAGTGAGCATTTCCTGTGATTTACGAAC	Subcellular localization
UBC11-104	CGGGGTACCATGGCGGCTACCATCAGC	CGCGGATCCGAGCATTTCTTGTGATTTCCTTAC	Spilt-YFP assay
UBC12-104	CGGGGTACCATGGCGACTGCCGCGAGCCA	CGCGGATCCGAGCATTTCCTGTGATTTACGAAC	Spilt-YFP assay
ABI3-106	CGCGGATCCATGGACGCCTCCGCCGGCT	ACGCGTCGACGATGCTAACCGCCATCTGGCTCATGG	Spilt-YFP assay
UBC11-AD/BD	CCGGAATTCATGGCGGCTACCATCAGCCA	CGCGGATCCTCAGAGCATTTCTTGTGATTTCCTTAC	Y2H assay
UBC12-AD/BD	CCGGAATTCATGGCGACTGCCGCGAGCCA	CGCGGATCCTCAGAGCATTTCCTGTGATTTACGAACAATGC	Y2H assay
DSG1-AD/BD	CCGGAATTCATGTCGGCCCCCGCGGCGGT	CGCGGATCCGACATACATGAACTCCCCACCCCTGAC	Y2H assay
UBC11-Spit-luc	ACGGGGTACCATGGCGGCTACCATCAGC	CGCGGATCCTCAGAGCATTTCTTGTGATTTCCTTAC	Spilt-LUC assay
UBC12-Spit-luc	ACGGGGTACCATGGCGACTGCCGCGAGCCA	CGCGGATCCTCAGAGCATTTCCTGTGATTTACGAACAATGC	Spilt-LUC assay
DSG1-Spit-luc	ACGGGGTACCATGTCGGCCCCCGCGGCGGT	ACGCGTCGACGACATACATGAACTCCCCACCCCTGAC	Spilt-LUC assay
ABI3-Spit-luc	CGCGGATCCAATGGACGCCTCCGCCGGCT	ACGCGTCGACGATGCTAACCGCCATCTGGCTCATGG	Spilt-LUC assay
q-ACTIN	CAGCACATTCCAGCAGAT	GGCTTAGCATTCTTGGGT	qRT-PCR

q-UBC11	GGTTCTCGGCTGGGTTAGT	AATGCATACACGTCCATCCG	qRT-PCR
q-UBC12	CGCCCGATACCCTGTATGAT	CGCAAGCTCATAACCGTTGG	qRT-PCR
q-LEA3	AGACCTCCAGCACGTCGCAG	GCCTGTTGGAGGACGCTGCC	qRT-PCR
q-RAB21	CAACGCTCCGGCAGCTCCAG	ATGCTGCTGCTCGCCCTTGT	qRT-PCR
q-ABI5	GGACGCAACTTTGGTTCCAT	GTCTACCACCTTGGCTTCCT	qRT-PCR
q-ABI3	AGCTCCGTATTATAACCGATGG	CAACCAAACCATGCGTACTTAA	qRT-PCR
q-SOS1	CTCCGTGCTCATAGAATCGC	ATACTCACTCAAGTGGGTCAATACC	qRT-PCR
q-NCED3	CCCCTCCCAAACCATCCAAACCGA	TGTGAGCATATCCTGGCGTCGTGA	qRT-PCR
q-NHX1	TGACCGTGAGGTTGCCCTTATGAT	AGAATACGGTGAGAATGCCGCTCA	qRT-PCR
q-HKT6	TGCATCACAGAACGGGACTC	AGCTGTAGCCAACCGAGAAC	qRT-PCR
q-ABI3-FLAG	GTAAGCATAAGCATGGCTCGC	ACCTTGCTTATCGTCATCGTCT	qRT-PCR
UBC11-HIS	CCGGAATTCATGGCGGCTACCATCAGCCA	CCGCTCGAGGAGCATTTCTTGTGATTTCCTTACAAGGC	Purification
UBC12-HIS	CCGGAATTCATGGCGACTGCCGCGAGCCA	CCGCTCGAGGAGCATTTCCTGTGATTTACGAACAATGCGT	Purification
HIS-UBC11	CCGGAATTCATGGCGGCTACCATCAGCCA	CCGCTCGAGTCAGAGCATTTCTTGTGATTTCCTTACAAGGC	Purification
HIS-UBC12	CCGGAATTCATGGCGACTGCCGCGAGCCA	CCGCTCGAGTCAGAGCATTTCCTGTGATTTACGAACAATGCGT	Purification
UBC11(92a)	TTTATCCGGATGGACGTGTAGCCATTTCTATTCTTCATCCGCC	GGCGGATGAAGAATAGAAATGGCTACACGTCCATCCGGATAAA	Purification
UBC12(92a)	TTTATCCTGATGGGCGCGTAGCCATTTCTATCCTTCATCCACC	GGTGGATGAAGGATAGAAATGGCTACGCGCCCATCAGGATAAA	Purification
GST-DSG1	CGCGGATCCATGTCGGCCCCCGCGGCGGT	ACGCGTCGACGACATACATGAACTCCCCACCCCTGAC	Purification
GST-ABI3	CGCGGATCCATGGACGCCTCCGCCGGCT	ACGCGTCGACGATGCTAACCGCCATCTGGCTCATGG	Purification
