# Supplementary material for: The E2 ubiquitin‐conjugating enzymes OsUBC11 and OsUBC12 promote rice seed germination by ubiquitinating and destabilizing OsABI3 in the ABA signaling pathway
Source: Plant J. 2026 Sep 10;127(5):e71096. doi: 10.1111/tpj.71096 (PMC13559216; doi:10.1111/tpj.71096)
Supplement: Supplementary file 2 [file TPJ-127-0-s002.pdf]

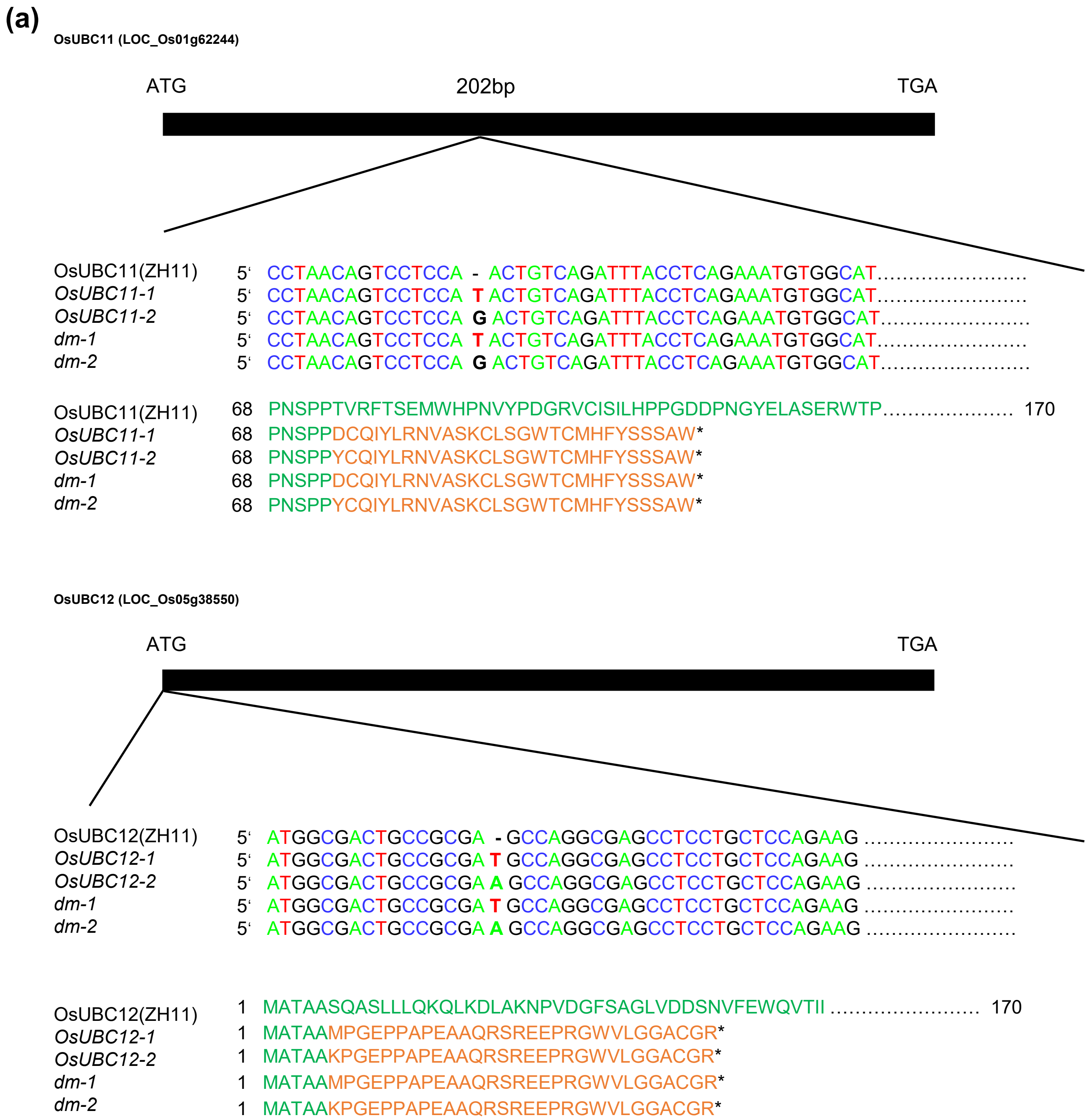

Figure S1. Characterization of the *OsUBC11/12* single and double mutants.
Independent mutant lines of the single and double mutants (*dm-1/-2*) are generated by CRISPR/Cas9 editing and their partial nucleotide and protein sequences are shown.

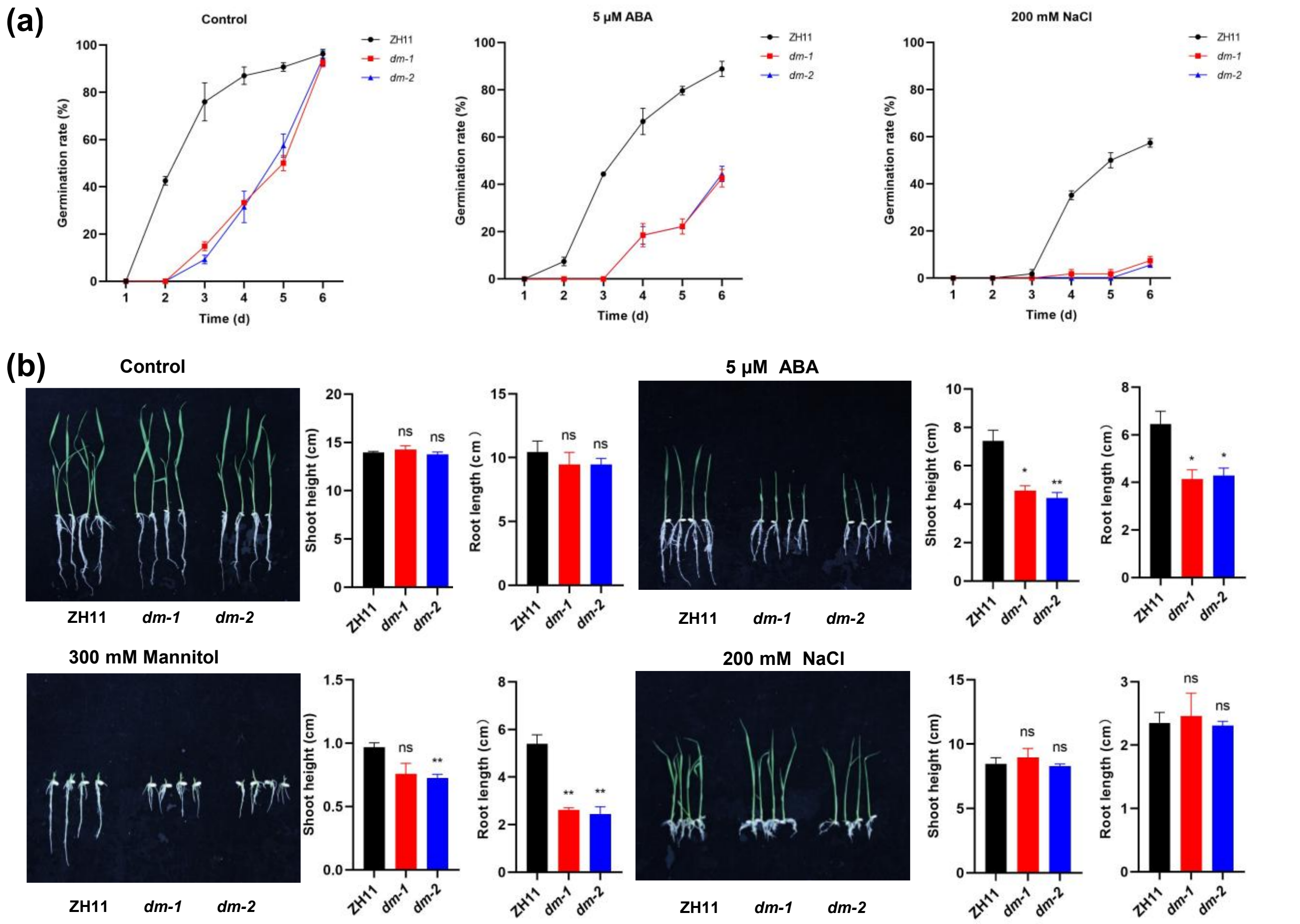

Figure S2. Regulation of seed germination and abiotic stress responses by Os*UBC11/12*.
(**a**) Germination assays. Wild-type (ZH11) and double mutants of *OsUBC11/12* (*dm-1/-2)* germinated on ½-MS agar medium containing water, ABA or NaCl, and their gemination rate was monitored. (**b**) Stress response assay. After germination, rice seedlings with uniform coleoptile and radicle lengths were selected and transferred to 1/2 MS medium (control) or supplemented with ABA, mannitol, or NaCl. The length of shoots and roots was measured. Data are presented as mean values ± SE, n=3. Asterisks indicate significance levels when comparing to ZH11 in *t*-test. (*, $P < 0.05$; **, $P < 0.01$; ns, not significant at $P < 0.05$).

(a)

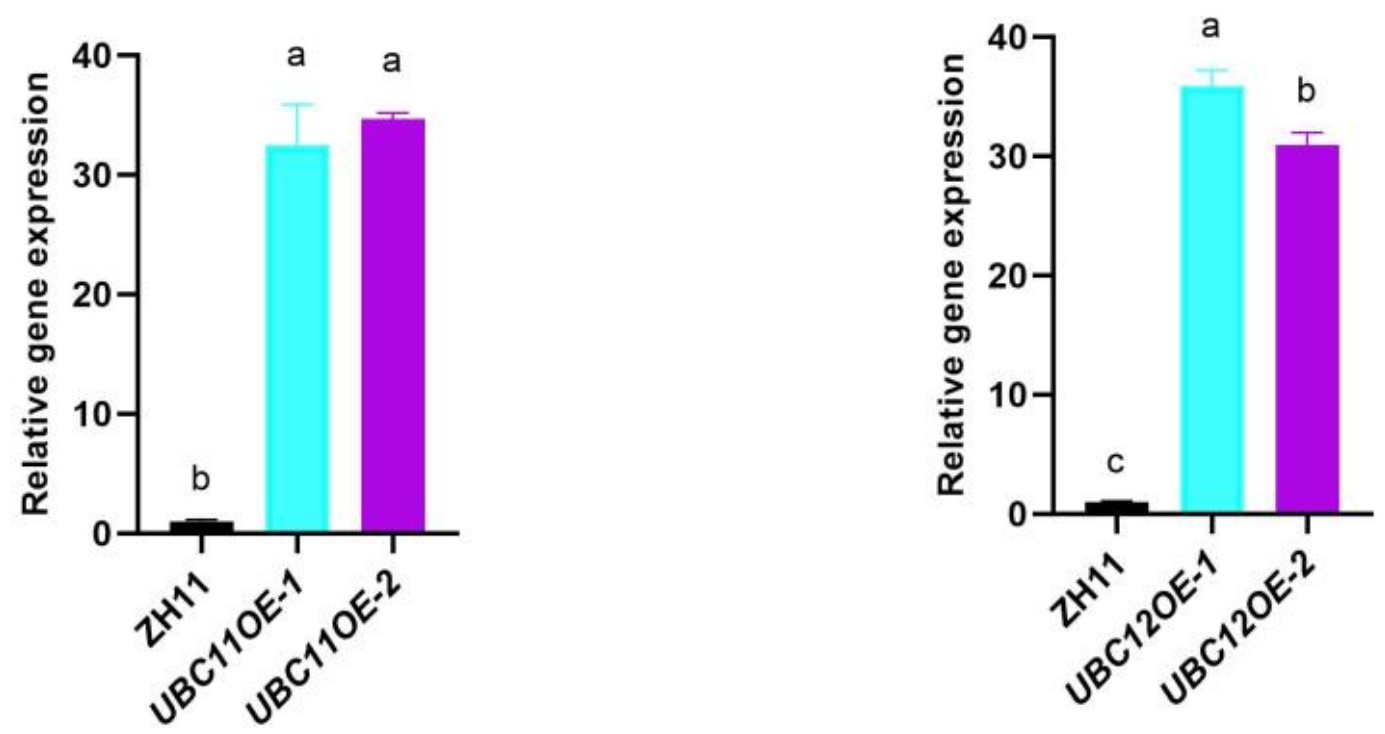

(b)

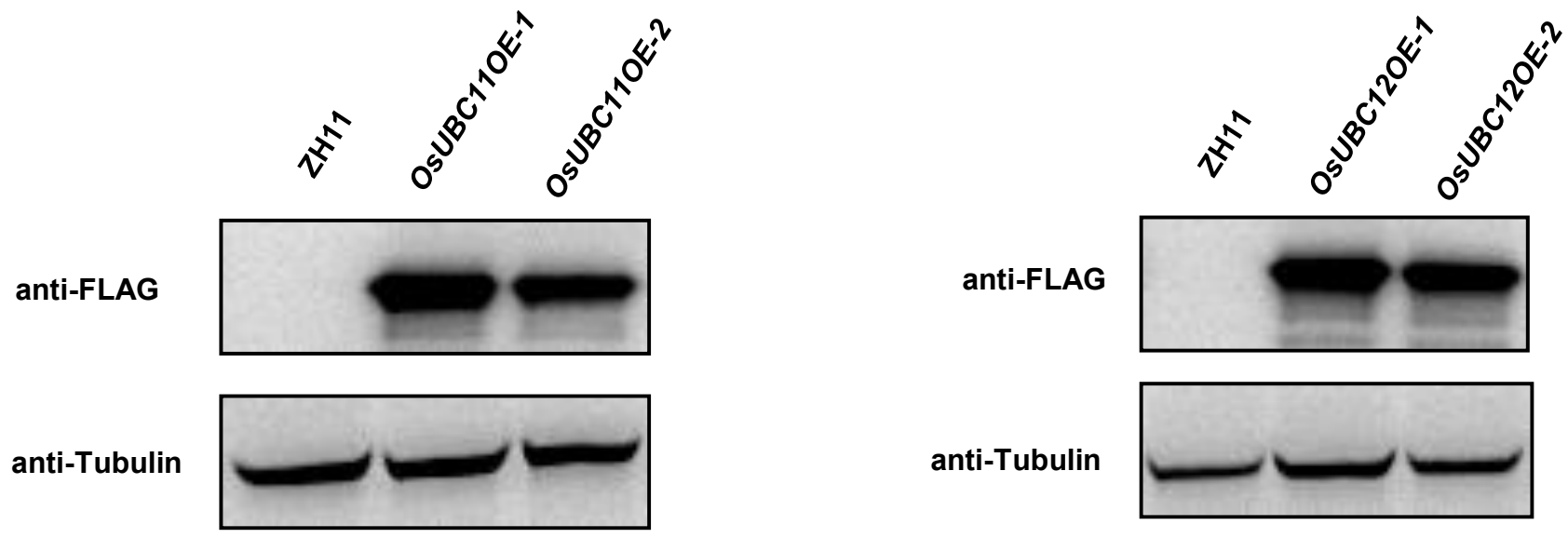

Figure S3. Validation of the transgene expression.
(**a**) Gene expression analysis. Total RNA were extracted from wild-type ZH11, *OsUBC11* overexpression plants (*OsUBC11OE-1/-2*), *OsUBC12* overexpression plants (Os*UBC12OE-1/-2*), and the expression level of *OsUBC11/12* was examined by RT-qPCR. Relative expression is the expression level of genes normalized to that of *Actin*. Error bars represent SE (n=3). Different letters indicate significant differences for comparisons between two samples as determined by LSD test following ANOVA analysis ($P<0.05$). (**b**) Western blotting analysis. Total proteins were extracted from ZH11, *OsUBC11OE-1/-2*, *OsUBC12OE-1/-2* and transgenic proteins were detected by western blotting using anti-FLAG antibody. Anti-Tubulin was used as a loading control.

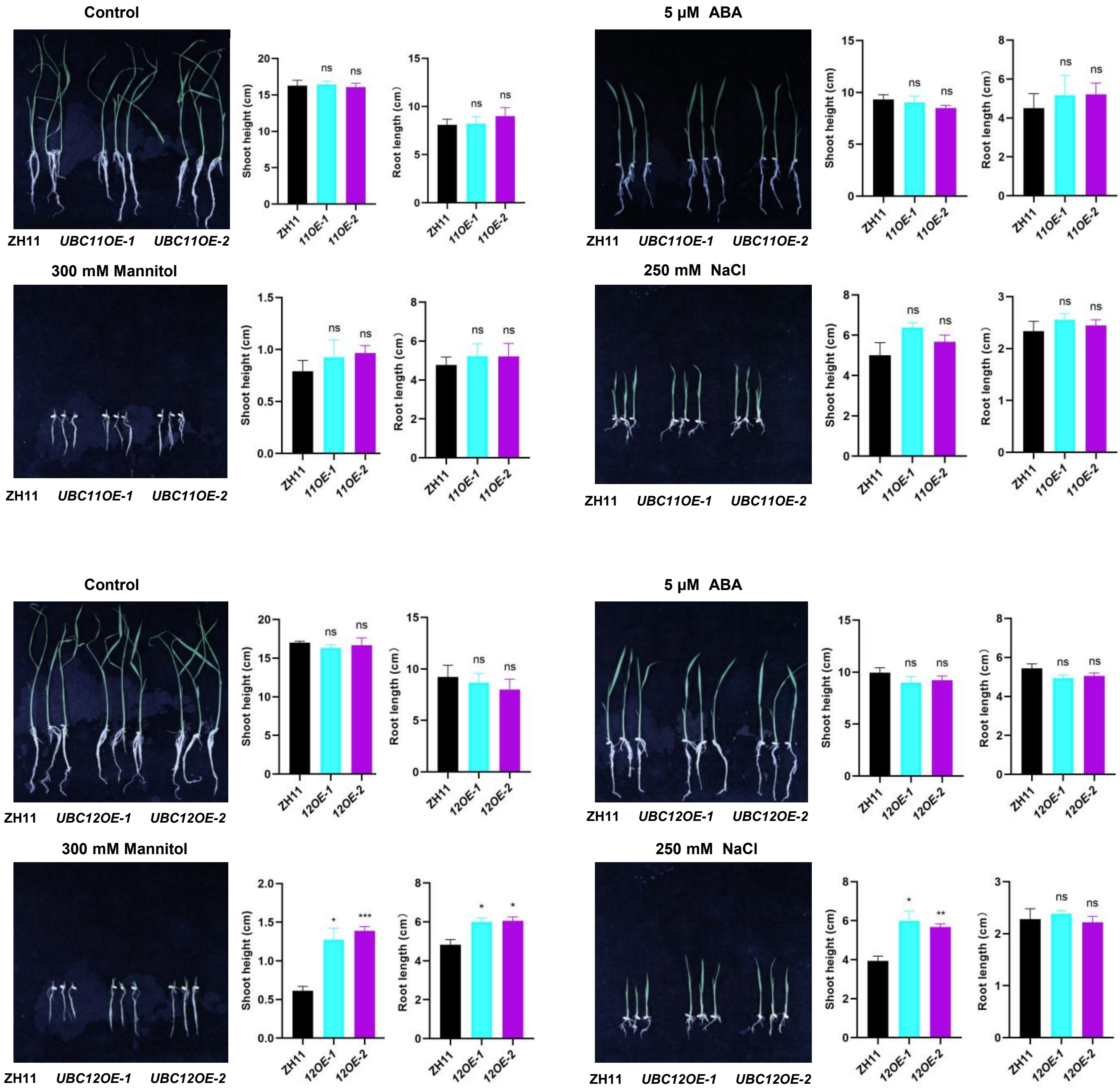

Figure S4. Regulation of seedling growth by Os*UBC11/12*.
Wild-type (ZH11) and *OsUBC11/12* overexpression seedlings with uniform coleoptile and radicle lengths were selected and transferred to grown in 1/2 MS medium containing water (control), ABA or NaCl, and their root/shoot length was measured. Data are presented as mean values ± SE, n=3. Asterisks indicate significance levels when comparing to ZH11 in *t*-test. (*, $P < 0.05$; **, $P < 0.01$; ***, $P < 0.001$; ns, not significant at $P < 0.05$).

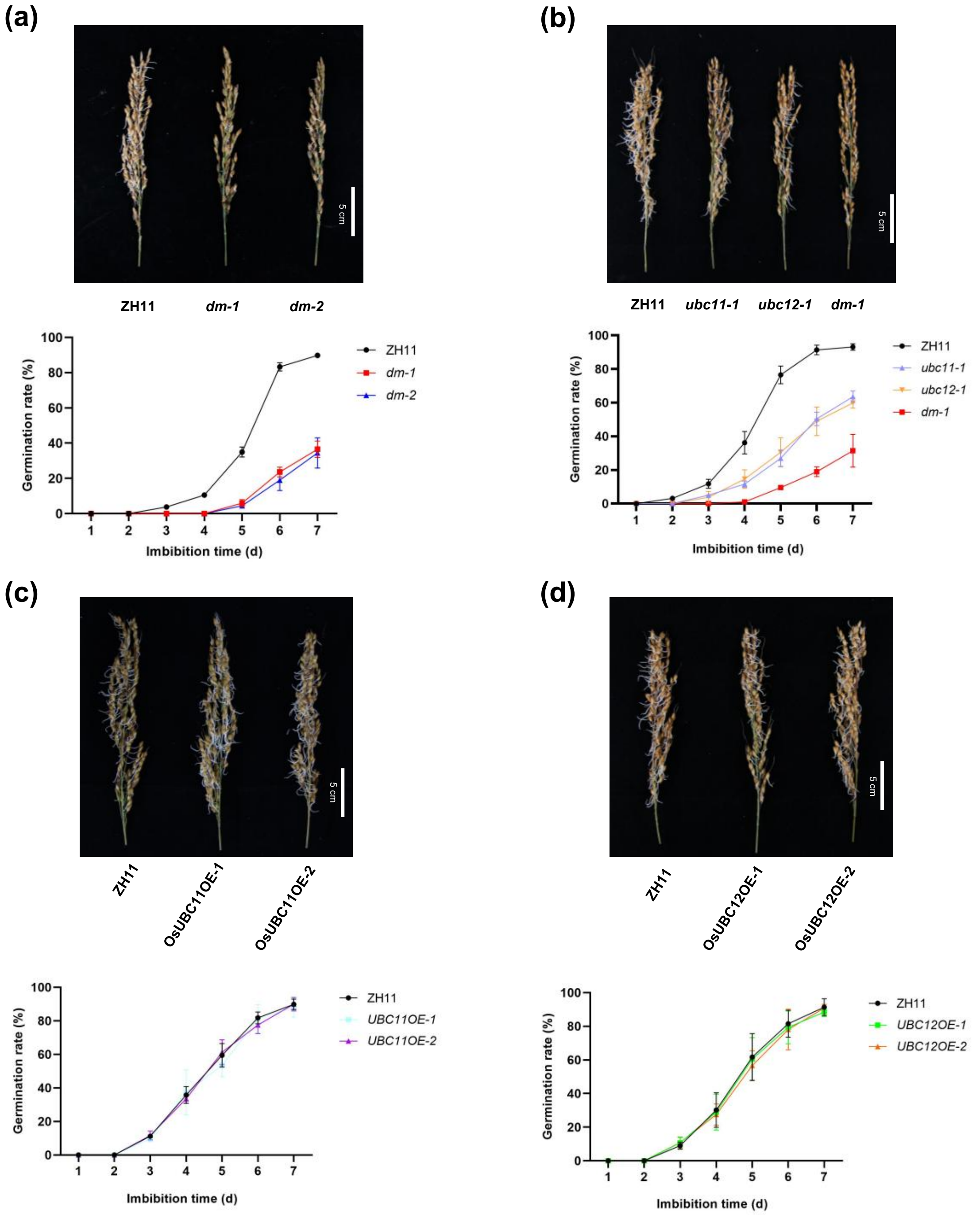

Figure S5. Regulation of pre-harvest sprouting by OsUBC11/12.

Panicles at a uniform maturity stage were selected from wild-type ZH11, *OsUBC11* single mutant (*ubc11-1*), Os*UBC12* single mutant (*ubc12-1*), and *OsUBC11/12* double mutant (*dm-1/-2*), *OsUBC11* overexpression plants (*OsUBC11OE-1/-2*), and *OsUBC12* overexpression plants (Os*UBC12OE-1/-2*), and immersed in water at 30°C for 7 days. Water was replaced every other day and germination was recorded. Data are presented as mean values +/- SE (n=3).

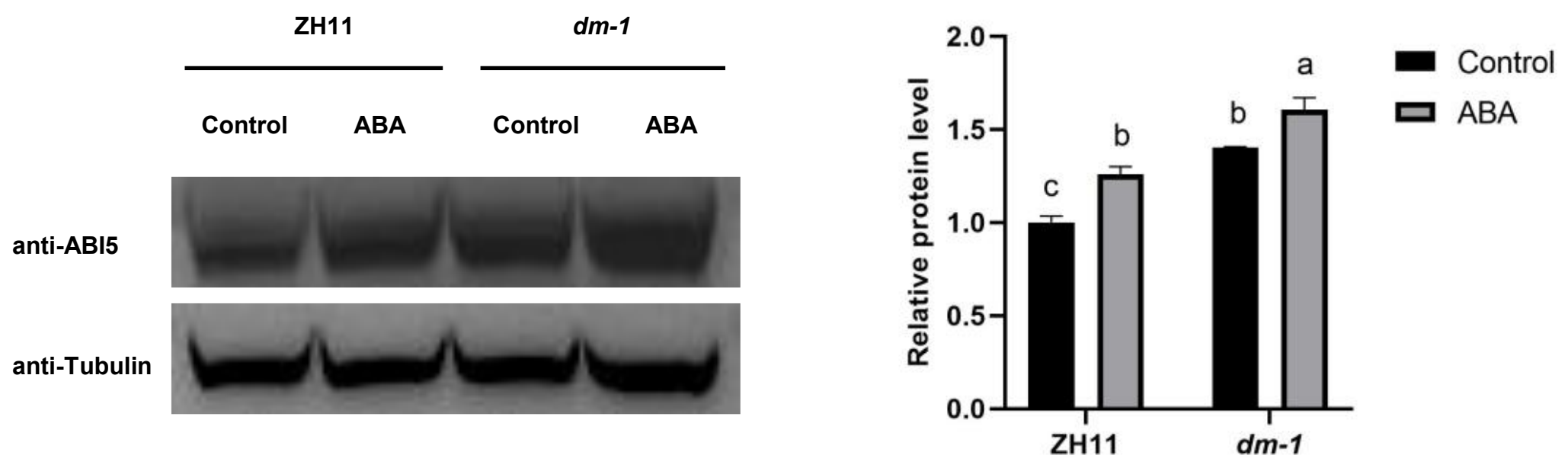

Figure S6. Regulation of OsABI5 by OsUBC11/12.
Total protein was extracted from ZH11 and the *OsUBC11/12* double mutant (*dm-1*) with or without ABA treatment, and endogenous OsABI5 protein levels were detected using an anti-ABI5 antibody. anti-Tubulin was used as a loading control. Error bars represent SE (n=3). Different letters indicate significant differences for comparisons between two samples as determined by LSD test following ANOVA analysis ($P<0.05$).

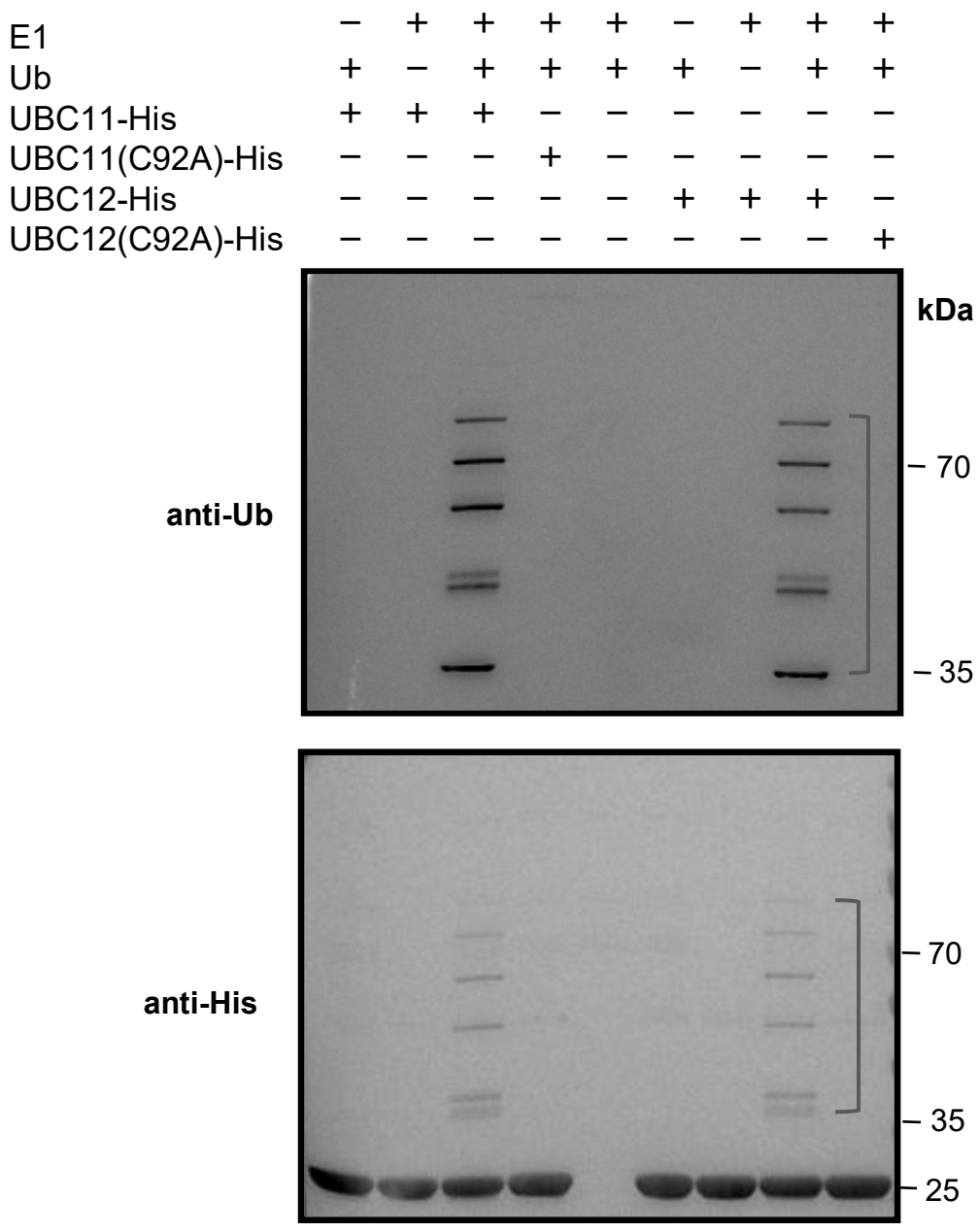

Figure S7. Auto-ubiquitination activity of OsUBC11/12.
Recombinant OsUBC11/12-His proteins or the mutated forms in the catalytic site were incubated in the presence or absence of ubiquitin (Ub) and E1, and checked with anti-ubiquitin and anti-His antibody. Brackets point to the ubiquitinated bands.

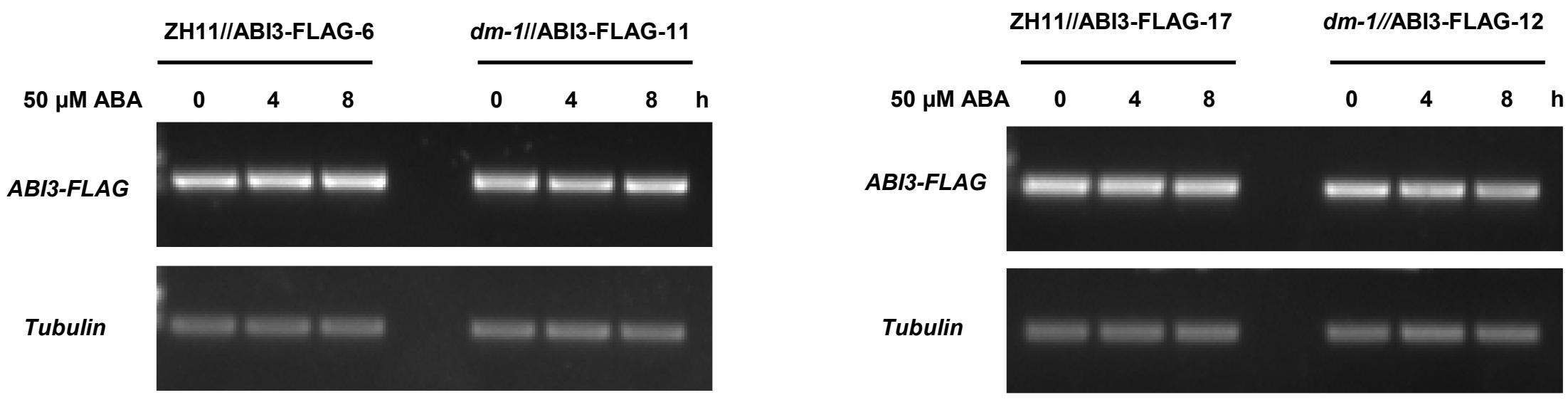

Figure S8. Validation of transgene expression.
ABI3-FLAG was overexpressed in wild-type ZH11 and *OsUBC11/12* double mutant (*dm-1*) background. One-week-old rice seedlings were treated with 50 μM ABA for 0, 4, and 8 h, and sampled for RT-qPCR. The expression level of the exogenous *ABI3-FLAG* was checked, and *Tubulin* was used as an internal control.

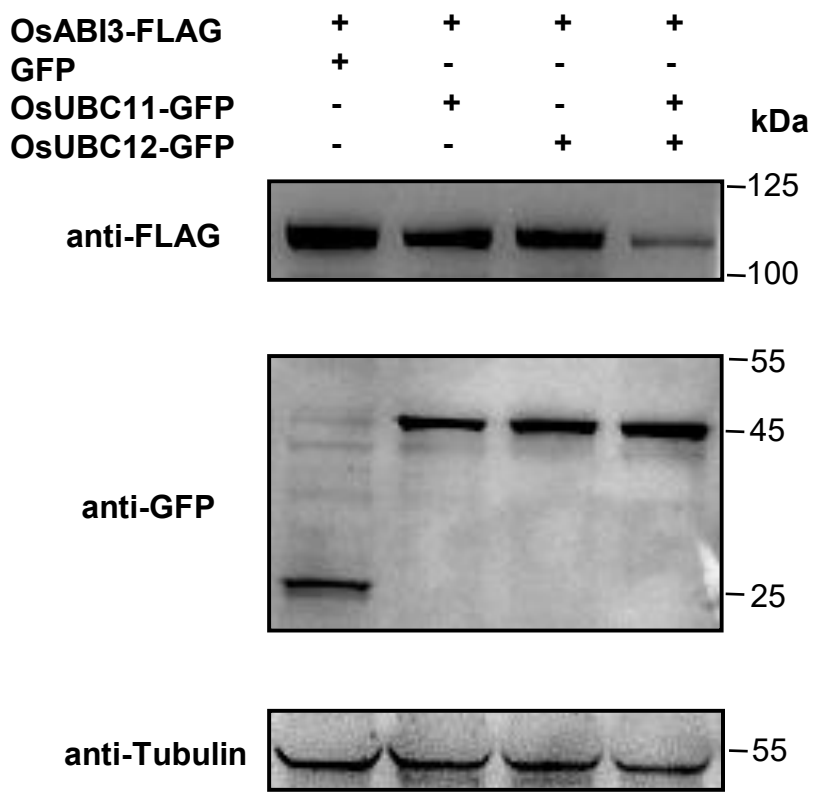

Figure S9. Regulation of OsABI3 accumulation by OsUBC11/12.
OsABI3 was co-expressed with GFP, OsUBC11-GFP alone or OsUBC12-GFP alone, or OsUBC11/12 together in tobacco leaves, and transgenic proteins were detected using anti-GFP or anti-FLAG. anti-Tubulin was used as a loading control.

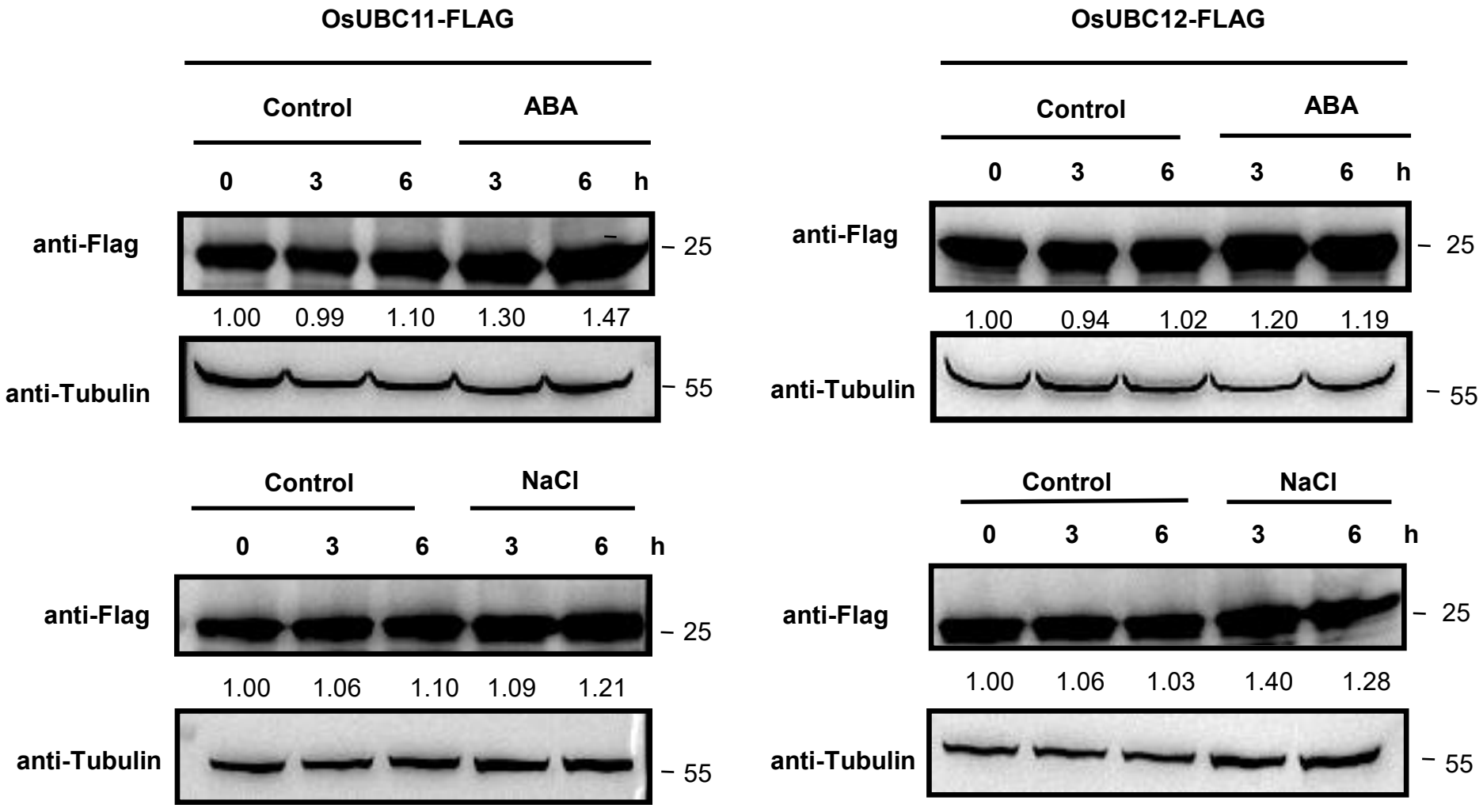

Figure S10. Accumulation of OsUBC11/12 proteins in response to ABA and NaCl treatment.
Seven-day-old wild-type ZH11, *OsUBC11/12-FLAG* overexpression plants driven by the constitutive *ubiquitin* promoter were subjected to ABA or NaCl treatment for 0, 3, and 6 h. Total proteins were extracted from leaves, and the transgenic protein level was detected by Western blotting using anti-FLAG antibody. The protein intensity was quantified and normalized to that of anti-Tubulin control. The relative protein intensity at 0 h was set to 1.00.

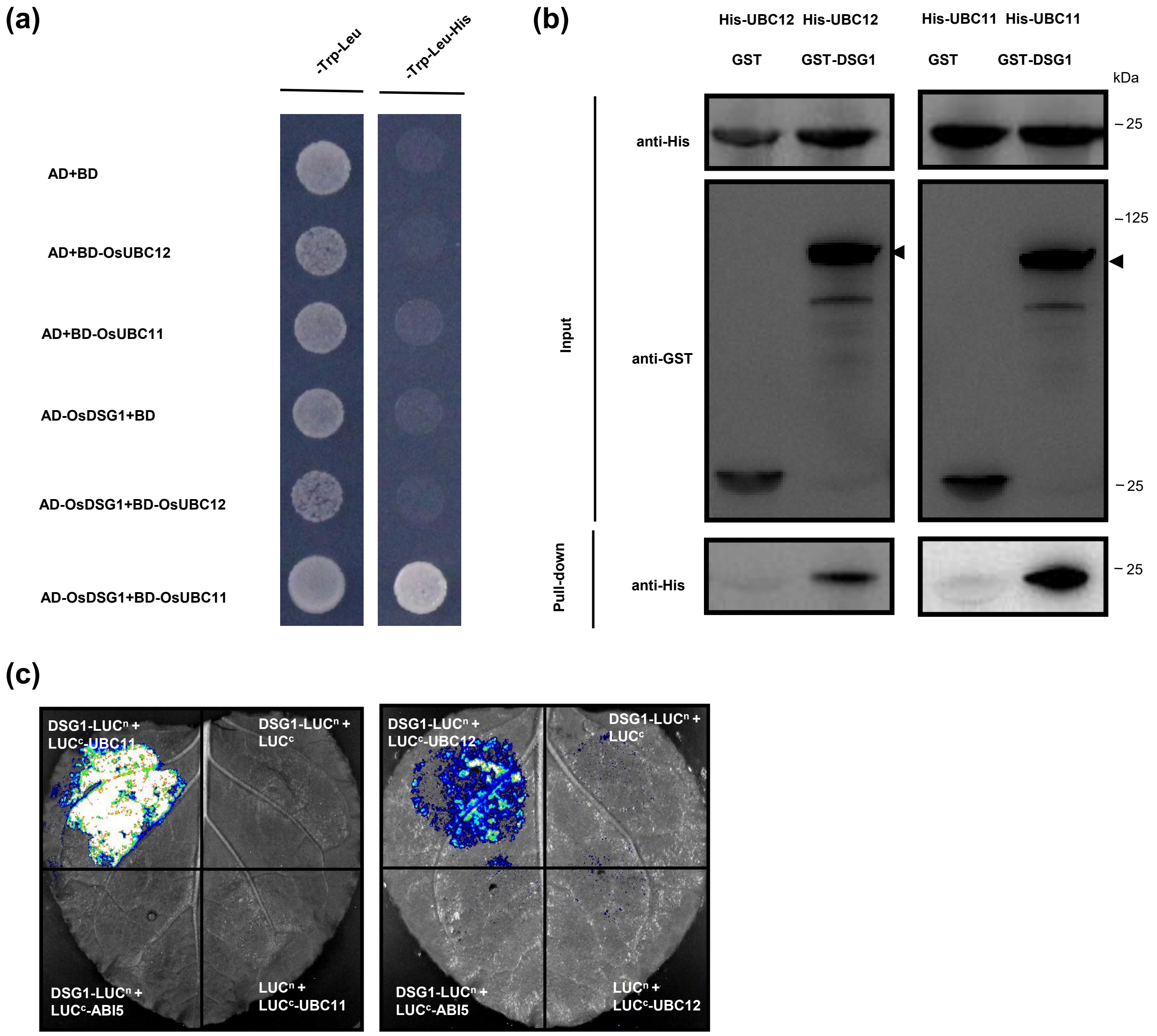

Figure S11. Interaction between OsUBC11/12 and OsDSG1.

(**a**) Yeast two-hybrid assays. The full-length of OsUBC11/12 were fused with the DNA binding domain (BD), while the full-length of OsDSG1 were fused with the activation domain (AD) of GAL4. HIS3 was used for interaction reporters. (**b**) Pull-down assay. OsDSG1 was fused with a GST tag, and OsUBC11/12 were fused with an His tag. After coincubation with both proteins, proteins were immunoprecipitated with Glutathione-Superflow resins and detected using anti-His antibody. Arrow head points to GST-DSG1. (**c**) Split-luciferase assays. OsDSG1 and OsUBC11/12 were fused with the N-terminal (nLUC) and C-terminal (cLUC) portion of firefly luciferase respectively. Different combinations of constructs were agro-infiltrated into tobacco leaves, and the chemiluminescence was observed.

OsSDG1 (LOC_Os09g26400)

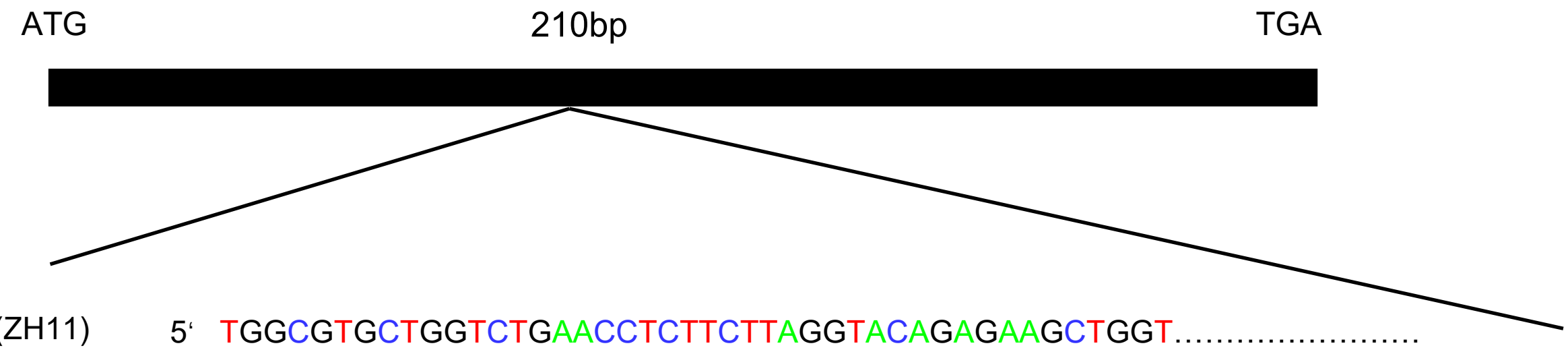

OsDSG1(ZH11)	5'	TGGCGTGCTGGTCTGAACCTCTTCTTAGGTACAGAGAAGCTGGT……………………
OsDSG1-1	5'	TGGCGTGCTGGTCTGAACCTCTT----------GTACAGAGAAGCTGGT ……………………
OsDSG1-2	5'	TGGCGTGCTGGTCTGAACCTCTTC--------GTACAGAGAAGCTGGT ……………………

OsDSG1(ZH11)	71	WRAGLNLFLGTEKLVTNPSEKEQLKTFILRAREHLDEKENEES………………… 320
OsDSG1-1	71	WRAGLNLLYREAGN*
OsDSG1-2	71	WRAGLNLFVQRSW*

Figure S12. Characterization of the *OsDSG1* single mutants.
Independent mutant lines of the single mutants are generated by CRISPR/Cas9 editing and their partial nucleotide and protein sequences are shown.

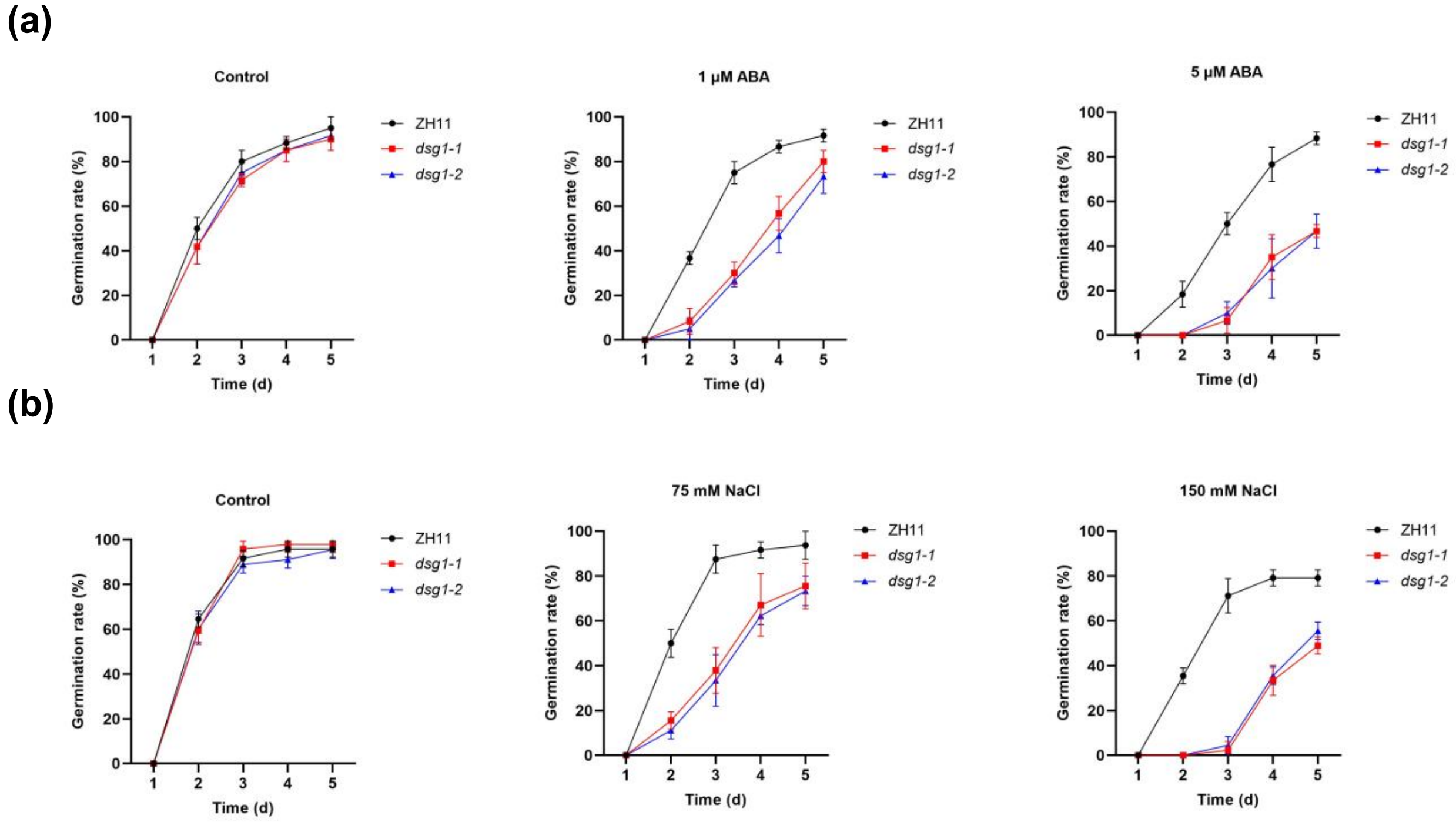

Figure S13. Phenotypic analysis of the *dsg1* mutants.
Wild-type ZH11 and *OsDSG1* mutants (*dsg1-1/-2*) were germinated on ½-MS agar medium containing ABA or NaCl, and the germination was monitored for 5 days. Values are means ± SE, n=3.

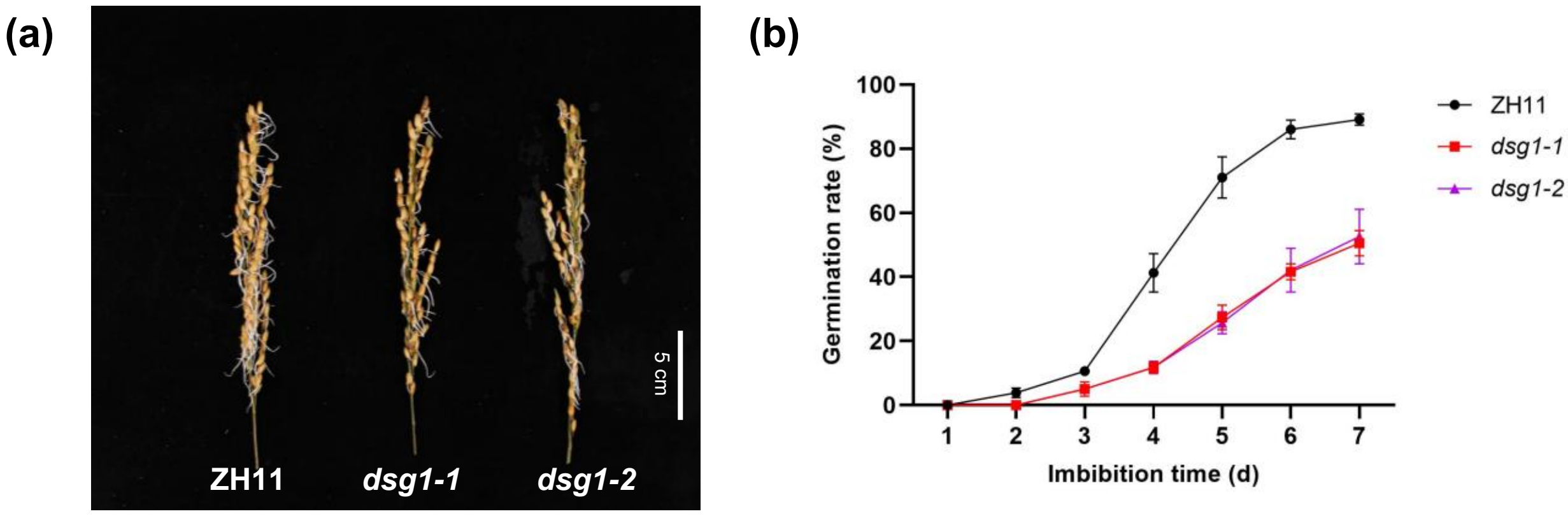

Figure S14. Regulation of pre-harvest sprouting by OsDSG1.
Panicles at a uniform maturity stage were selected from wild-type ZH11 and *OsDSG1* mutant (*dsg1-1/-2*), and immersed in water at 30°C for 7 days. Water was replaced every other day and germination was recorded. Data are presented as mean values +/- SE (n=3).
